# Evaluation of the reproducibility and performance characteristics of the Phagomagnetic separation-qPCR assay for rapidly detecting viable *Mycobacterium avium* subsp. *paratuberculosis* in bovine milk and feces

**DOI:** 10.3389/fvets.2025.1677096

**Published:** 2025-12-15

**Authors:** Irene R. Grant, Iker A. Sevilla, Elena Molina, Beatriz Romero Martinez, Víctor Lorente-Leal, Virginie C. Thibault-Poisson, Martina Cechova, Heike Köhler

**Affiliations:** 1 School of Biological Sciences, Queen's University Belfast, Belfast, United Kingdom; 2 Rapid-Myco Technologies Limited, Belfast, United Kingdom; 3 Departamento de Sanidad Animal, NEIKER-Instituto Vasco de Investigación y Desarrollo Agrario, Basque Research and Technology Alliance (BRTA), Derio, Bizkaia, Spain; 4 Centro de Vigilancia Sanitaria Veterinaria (VISAVET), Universidad Complutense de Madrid, Madrid, Spain; 5 Departamento de Sanidad Animal, Facultad de Veterinaria, Universidad Complutense de Madrid, Madrid, Spain; 6 Departamento de Genética, Fisiología y Microbiología, Facultad de Ciencias Biológicas, Universidad Complutense de Madrid, Madrid, Spain; 7 ANSES, Ploufragan-Plouzané-Niort Laboratory, Ruminant Diseases and Welfare Unit, Niort, France; 8 GDS France (National Animal Health Farmers' Organization), Paris, France; 9 Department of Microbiology and Antimicrobial Resistance, Veterinary Research Institute, Brno, Czechia; 10 Institute of Molecular Pathogenesis, Friedrich-Loeffler Institut, Jena, Germany

**Keywords:** inter-laboratory trial, *Mycobacterium avium* subsp. *paratuberculosis* (MAP), bovine milk, bovine feces, Phagomagnetic separation (PhMS)-qPCR assay, viability assay

## Abstract

Inter-laboratory trials were carried out to evaluate the reproducibility and estimate test performance characteristics of the Phagomagnetic separation (PhMS)-qPCR assay, a novel phage-based assay recently developed as a rapid alternative to culture for detecting viable *Mycobacterium avium* subsp. *paratuberculosis* (MAP) in bovine milk and feces. Unique reagents and a detailed instruction manual required for the PhMS-qPCR assay were provided to five European veterinary diagnostic laboratories by Rapid-Myco Technologies Limited. Milk and feces test panels were prepared at NEIKER and distributed to participant laboratories between April–June 2023 and March–May 2024, respectively. Each test panel comprised of MAP-spiked and/or naturally infected bovine milk or feces samples (18 samples per panel on two separate occasions for each sample matrix). The six participant laboratories (including organizer) performed automated or manual PhMS and used whatever qPCR instrument they had available. All laboratories used the IDEXX RealPCR MAP DNA test for the qPCR part of the assay. Generally, substantial agreement was observed overall between PhMS-qPCR results and reference culture results for spiked milk (Kappa value 0.5982) and naturally MAP-infected feces (Kappa value 0.7780 using an amended protocol). Preliminary estimates of the detection (analytical) sensitivity (Se), detection specificity (Sp) and trueness (T) of the PhMS-qPCR assay applied to bovine milk and feces were obtained. The mean Se, Sp, and T values across six laboratories were 93.1%, 67.9%, and 88.7% when milk was tested and 84.1%, 93.7%, and 88.9% when feces was tested. Overall, the PhMS-qPCR assay performed well in multiple laboratories and test reproducibility was demonstrated (Cohen's Kappa ≥0.6–1.000). The estimates of performance characteristics of the PhMS-qPCR assay are generally acceptable for a potential diagnostic test. Hence the PhMS-qPCR assay shows considerable promise as a rapid test to detect viable MAP in veterinary specimens such as milk and feces. Further and fuller validation of the assay will continue to assess its diagnostic potential.

## Introduction

Diagnosis of Johne's disease (JD, also known as Paratuberculosis) requires the demonstration of a humoral immune response to *Mycobacterium avium* subsp. *paratuberculosis* (MAP) by serum or milk ELISA, or detection of MAP DNA in feces by a molecular method such as PCR/qPCR, or isolation of MAP from feces by culture (1, 2). Culture is the gold standard method of detecting viable MAP but is tedious to perform and slow to provide results; primary isolation can take 12–20 weeks (3), which prevents timely decision making for JD control purposes.

The development of more rapid methods to specifically detect viable MAP in veterinary specimens such as milk and feces, as an alternative to, or to potentially replace, slow culture methods for diagnostic purposes, has been the focus of MAP researchers over recent decades. Such methods include viability PCR involving propidium monoazide dye and UV cross-linking (4), and mycobacteriophage-based methods [see review by Grant (5)]. The latter include the phage-PCR assay (6), the Peptide-mediated magnetic separation (PMS)-phage assay (7–10), the Actiphage^^®^^ Rapid assay (11), and the Phagomagnetic separation (PhMS)-qPCR assay (12, 13). There appears to be considerable interest amongst MAP researchers in adopting these rapid phage-based methods to assess MAP viability, instead of culture. For example, Steuer et al. (14) used the PMS-phage amplification assay (9) as a research tool to determine the effect of copper ions on the viability of MAP cells. Beinheurova and Slana (15, 16) applied the Actiphage^^®^^ Rapid assay to test fresh cow, sheep and goats' milk and environmental samples. Hosseiniporgham et al. (17) attempted to recreate the PhMS-qPCR assay to test for viable MAP in unpasteurized sheep and goats' milk. A recent review by Martins et al. (18) listed phage-based methods as a new diagnostic for JD but highlighted that there was a knowledge gap in relation to test characteristics (sensitivity and specificity) when applied to feces and blood.

The PhMS-qPCR assay is a novel patented method (EP4022095C0; US2023340617A1; WO2021058606A1) for detection of viable MAP developed at Queen's University Belfast by Foddai and Grant (12). The test has been demonstrating good sensitivity and specificity for this purpose in the test development (progenitor) laboratory—sensitivity 70.1% and specificity 96.3% determined by Bayesian analysis of milk testing results (13). It has more recently been optimized for testing for viable MAP in bovine feces and blood (19); no specificity or sensitivity estimates are available yet for the latter two sample types. If the PhMS-qPCR assay is going to progress to become a commercial diagnostic test, it will need to be successfully transferred to other potential end-user (adopter) laboratories and give similar results, i.e., be reproducible (20). Inter-laboratory trials may be undertaken as part of test development and validation and are organized and reviewed as discrete events to compare results obtained by different laboratories on the same set of samples (21). The World Organization for Animal Health (WOAH) validation pathway for new veterinary diagnostic tests recommends that reproducibility of a new test be assessed by having three or more laboratories involved in a multi-laboratory trial (20, 22).

Further validation of the PhMS-qPCR assay is the subject of the study reported here. The aims of this inter-laboratory study were to, firstly, evaluate if the novel PhMS-qPCR assay for detecting viable MAP in milk and feces could be successfully transferred to several different European veterinary diagnostic laboratories (reproducibility), and, secondly, to preliminarily assess the performance of the test (detection specificity, detection sensitivity and trueness) in comparison with culture (the only other method capable of detecting only viable MAP) using naturally and artificially contaminated milk and feces samples. The results of milk and feces inter-laboratory trials are presented here.

## Materials and methods

### Participating laboratories

Rapid-Myco Technologies Limited (a spin-out company from Queen's University Belfast) is the test progenitor laboratory, and the other participating laboratories were performing the PhMS-qPCR assay for the first time. Dr Iker Sevilla, NEIKER, was contracted by Rapid-Myco Technologies Limited to set up and coordinate the milk and feces trials, source naturally infected milk and feces samples, spike milk and feces samples with MAP, distribute test panels to participating laboratories, and to collate PhMS-qPCR results from each laboratory. Five European laboratories with prior experience of MAP testing and/or magnetic separation (of bacterial cells or DNA) were recruited, in addition to the originator laboratory, to participate in a milk trial from April–June 2023 and in a subsequent feces trial from March–May 2024. The identities and roles of the six laboratories involved in this study are provided in Table 1. All laboratories participated voluntarily and were reimbursed the cost of the IDEXX RealPCR MAP DNA test kits required (where this was permitted) in exchange for their participation.

**Table 1:** Roles and responsibilities of participant laboratories.

**Activity**	**RAPID-MYCO (UK)**	**NEIKER (ES)**	**VISAVET (ES)**	**ANSES (FR)**	**VRI (CZ)**	**FLI (D)**
High level planning of milk and feces trials	✓	✓	×	×	×	×
Provision of PhMS reagents and detailed test instructions	✓	×	×	×	×	×
Sourcing of naturally infected milk and feces	×	✓	×	×	×	×
MAP spiking of milk and feces	×	✓	×	×	×	×
Confirmation of MAP spiking levels and/or presence or absence of viable MAP in test samples	(✓)^*^	✓	×	(✓)^*^	×	(✓)^*^
Coordination of milk and feces trials and distribution of test panels to participants	×	✓	×	×	×	×
Milk and feces testing (two test panels for each sample matrix)	✓	✓	✓	✓	✓	✓
Return of PhMS-qPCR results to Iker Sevilla, NEIKER	✓	✓	✓	✓	✓	✓
Collation of trial results and return to Rapid-Myco	×	✓	×	×	×	×
Analysis of trial results	✓	×	×	×	×	×
Review/approval of report on milk and feces trials	✓	✓	✓	✓	✓	✓

^*^These laboratories carried out DNA extraction and qPCR or HPC decontamination and culture on replicate set(s) of test panel samples to demonstrate presence of MAP DNA or viable MAP. However, NEIKER established the Reference Value (“MAP positive” or “MAP negative”) by culture for each sample tested, against which PhMS-qPCR results were compared during data analysis and for calculation of test performance characteristics.

**Figure 1:**
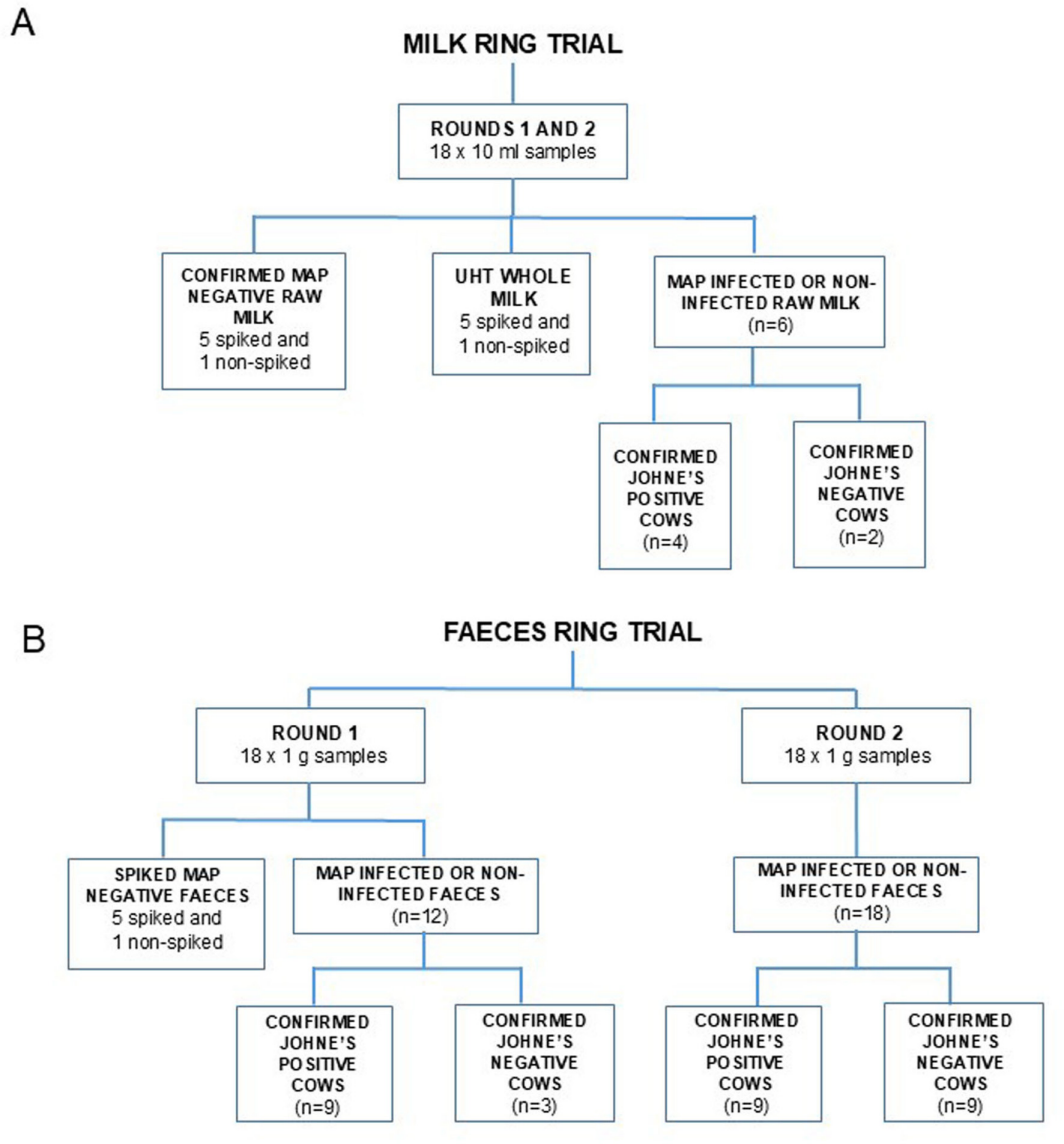
Composition of test panels of 18 samples distributed to participating laboratories during **(A)** milk trial and **(B)** faeces trial. Both spiked and naturally infected milk and feces samples were confirmed as either “MAP positive” or “MAP negative” by parallel MAP-specific PCR after DNA extraction, and HPC decontamination and culture to confirm if viable MAP were present.

In advance of the milk and feces trials commencing, each participating laboratory was supplied with a detailed step-by-step instruction manual for the PhMS-qPCR assay by Rapid-Myco Technologies Limited, however no in-person training in relation to the new method took place. This was to mimic the situation where laboratory personnel were applying the test in an adopting laboratory for the first time, interpreting the instruction manual themselves, and using whatever magnetic separation (automated or manual) and other required equipment (e.g., qPCR machine, incubator, centrifuge) they had available. Prof. Grant responded to any queries in connection with applying the PhMS-qPCR assay that any participant had once they had reviewed the instructions and before test panels were distributed. Each trial (milk or feces) comprised of two test panels (termed Rounds 1 and 2 herein), distributed on separate dates, a few weeks apart. The composition of each test panel was as shown in Figure 1.

### Preparation of MAP inoculum for spiking and sourcing of naturally infected samples

NEIKER personnel grew MAP strain ATCC 19698 in Middlebrook 7H9 broth supplemented with 10% OADC (both Becton Dickinson, M7H9-OADC) and 2 μg/ml mycobactin J (ID-Vet) for 4 weeks at 37 °C without shaking. The culture was sonicated at 37 KHz for 4 min in pulse mode in a sonicator bath to disperse clumps (23) and OD_600nm_ adjusted to 0.1 (10^6^-10^7^ cells/ml), before a 10-fold dilution series was prepared in M7H9-OADC. MAP inoculum was cultured on M7H9-OADC-mycobactin agar plates to obtain an indication of how many MAP had been added to milk or feces samples (see Supplementary Table S1 for MAP levels achieved in Round 1 and Round 2 milk samples). Target spiking levels with MAP ATCC 19698 strain were 10^5^, 10^4^, 10^3^, 10^2^, and 10^1^ MAP/ml milk or/g feces.

Commercial UHT milk was purchased from a supermarket near NEIKER, Spain. MAP-negative raw milk was obtained from the bulk tank of a Paratuberculosis-free bovine herd from the Basque Country, Spain. Raw milk from Paratuberculosis-affected cows (ELISA and/or fecal PCR positive) was collected directly from six lactating cows belonging to a herd in the Basque Country, Spain, which was under surveillance due to PTB problems. All raw milk samples were aliquoted into 15 ml centrifuge tubes (9 ml if sample was to be spiked, 10 ml for naturally infected milks) soon after collection and stored frozen (−80 °C) until spiking and/or shipping. UHT milk was aliquoted the day of sample preparation and distribution. The day before shipping, frozen raw milk samples were placed at 4 °C to allow them to thaw by next morning. On the morning of shipping day, an OD_600nm_ 0.1 MAP suspension was prepared and used to make sufficient volume of four consecutive 10-fold dilutions in M7H9-OADC broth (10^−1^, 10^−2^, 10^−3^, and 10^−4^ containing 10^5^-10^6^, 10^4^-10^5^, 10^3^-10^4^, and 10^2^-10^3^ MAP/ml, respectively) for spiking purposes. UHT milk and confirmed MAP-negative raw milk aliquots (9 ml) were spiked with 1 ml of the MAP dilutions.

For the Milk Trial, the milk sample volume selected for the trial was 10 ml milk, rather than the more usual 50 ml milk sample test volume, for logistical and cost reasons. The 18 samples in each milk test panel comprised of five MAP-spiked (6 × 10^5^-6 × 10^1^ CFU/ml milk for Round 1 and 3 × 10^6^-3 × 10^2^ CFU/ml milk for Round 2) and one non-spiked UHT milk, five MAP-spiked (6 × 10^5^-6 × 10^1^ CFU/ml milk for Round 1 and 3 × 10^6^-3 × 10^2^ CFU/ml milk for Round 2) and one non-spiked raw milk, and raw milks from six Paratuberculosis-affected cows (Figure 1A and Supplementary Table S1). NEIKER staff spent considerable time prior to the Milk Trial commencing trying to identify confirmed MAP positive cows shedding detectable numbers of MAP in their milk; an exercise which proved difficult. Ultimately, the milk from four cows that demonstrated low levels of MAP by direct qPCR but not by HEYM culture (cows 2, 4, 5. and 6), and two cows that had no detectable MAP by either qPCR or HEYM culture (cows 1 and 3), were included in the test panels for Rounds 1 and 2 (Supplementary Table S2). The high Cq values observed by NEIKER staff indicate low levels of MAP contamination, probably in the range 1–100 cells/10 ml milk, but no convincing evidence of viable MAP. In contrast, the presence of varying levels of MAP in the spiked raw and UHT milk samples was confirmed at NEIKER (Supplementary Table S1) by direct qPCR and HEYM culture (using methodology described below).

The test panels for both rounds of the milk trial were the same, whereas the 18 samples in the feces test panels differed between rounds. For Round 1 of the feces trial, the panel comprised five MAP-spiked (10^5^-10^6^, 10^4^-10^5^, 10^3^-10^4^, 10^2^-10^3^, and 10^1^-10^2^ MAP cells/g) plus one non-spiked feces samples and 12 naturally infected feces samples of known MAP status (nine from confirmed MAP positive cows and three from confirmed MAP negative cows). For Round 2, the panel comprised of only naturally infected feces, nine from confirmed MAP positive cows (eight of which had formed part of Round 1 test panel) and nine from confirmed MAP negative cows (three of which had formed part of Round 1 test panel; Figure 1B). Feces samples supplied for testing were all 1 g aliquots in 15 ml centrifuge tubes. Samples were blind-coded and shipped refrigerated to each participating laboratory by overnight courier.

### Confirmation of presence of MAP in test samples by culture and qPCR at NEIKER

A replicate set of samples from each test panel was subjected to culture and MAP-specific qPCR at NEIKER to generate reference results against which PhMS-qPCR results from all participating laboratories would be compared during data analysis and calculation of test performance characteristics. The spiked and naturally infected milks (10 ml) and spiked and naturally infected feces (1 g) were subjected to culture to confirm the presence of viable MAP and to DNA extraction followed by MAP-specific qPCR to demonstrate the presence or absence of MAP DNA.

Milk culture was performed as follows: after incubation at room temperature for 1 h and at 35–37 °C for 10–15 min, milk samples (10 ml) were centrifuged for 15 min at 2,500 × g and the supernatants discarded. Pellets from raw and naturally infected milk samples were resuspended in 5 ml 0.75% hexadecylpyridinium chloride (HPC, Sigma-Aldrich) and decontaminated at room temperature for 5 h. After decontamination, samples were centrifuged again for 15 min at 2,500 × g and the supernatants were discarded. UHT milk pellets were not decontaminated but simply centrifuged. The pellets obtained from both UHT milk and decontaminated raw milk test portions were resuspended with 200 μl phosphate buffered saline containing 0.05% Tween 20 (PBST, Sigma-Aldrich) before being distributed onto two slants of commercial Herrold's egg yolk mycobactin medium supplemented with Amphotericin B (50 mg/L), Nalidixic acid (50 mg/L), Vancomycin (50 mg/L), and sodium pyruvate (4 g/L; HEYM/ANV; Becton Dickinson, United States). Fecal culture was performed as follows: feces samples were prepared in the same manner as for the PhMS-qPCR assay to second centrifugation step. The fecal pellet was resuspended in 0.75% HPC and incubated at room temperature for 24 h. The samples were centrifuged and pellets resuspended in PBS before being inoculated onto one slant of HEYM/ANV medium. All HEYM cultures were incubated at 37 °C ± 1 °C for up to 18 weeks and examined periodically for evidence of colonies typical of MAP. Any colonies obtained were confirmed as MAP by qPCR.

Direct PCR testing of spiked milk and feces samples was carried out to confirm that target MAP spiking levels had been achieved. For milk, ADIAPURE™ PARATB DNA extraction (ADIAGENE, France) of 10 ml spiked milks was performed according to the manufacturer's instructions, followed by IDEXX RealPCR MAP DNA test, and for spiked feces a MagMAX total nucleic acid isolation kit (AM1840, Ambion, Applied Biosystems, Foster City, CA, USA) was employed for DNA extraction followed by IDEXX RealPCR MAP DNA kit (Rounds 1 and 2) and IS900/ISMAP02 qPCR (9) (Round 2 only). Naturally infected milk and feces samples were similarly tested by qPCR to confirm presence or absence of MAP infection.

Supplementary Tables S1–S4 summarize the reference culture results (plus parallel direct qPCR results) obtained by NEIKER, which established the viable MAP status of the spiked and naturally infected milk and feces samples that were tested by participating laboratories during the inter-laboratory trials.

### Milk and feces sample preparation in participating laboratories before PhMS-qPCR testing

**Figure 2:**
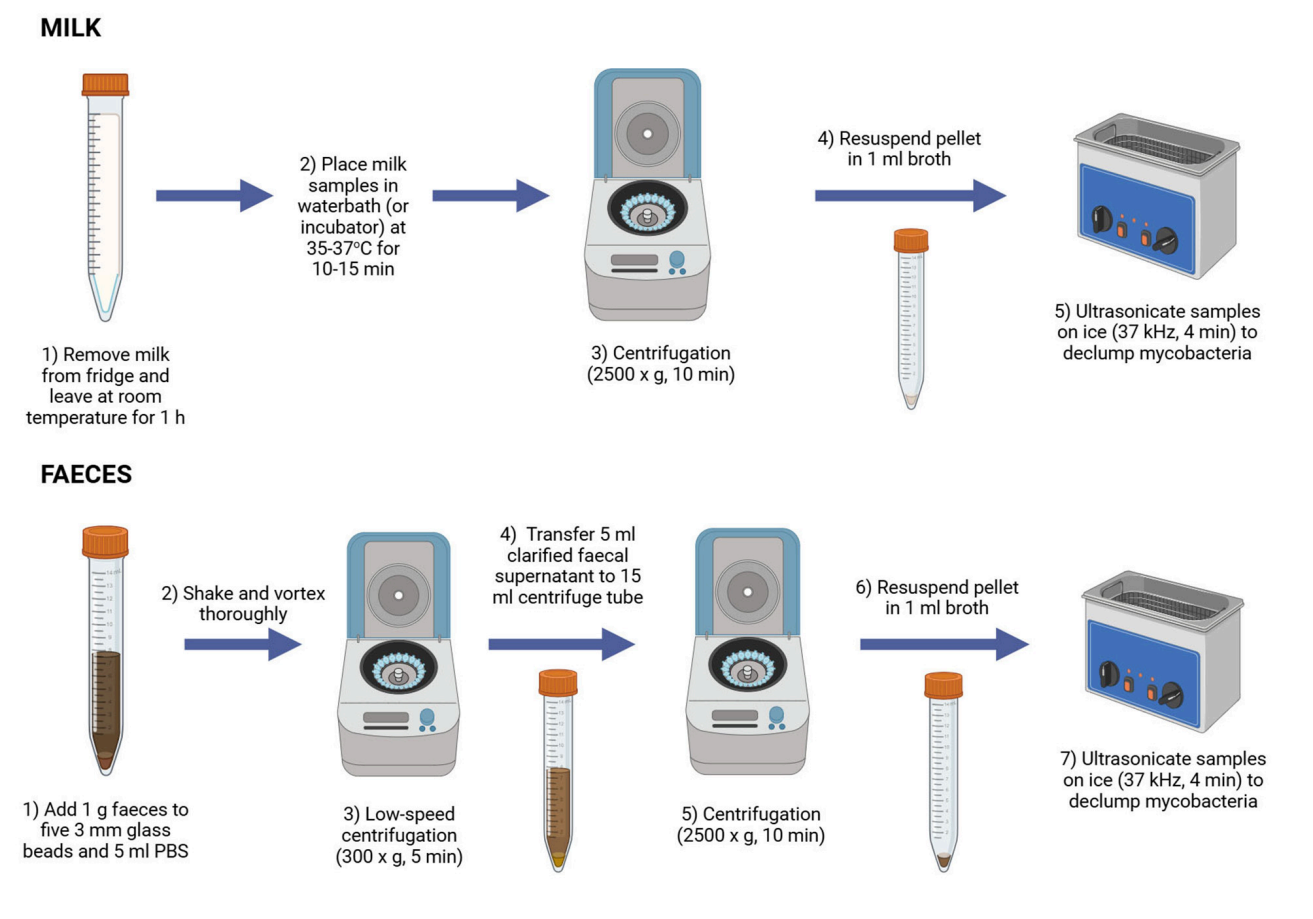
Schematic showing steps involved in milk and feces sample preparation prior to application of automated or manual PhMS (Created in https://BioRender.com).

Participating laboratories were supplied with three sets of the 18 test samples for each round of the trials and were expected to test one set by PhMS-qPCR and, if practicable, a second set by culture or direct qPCR. Samples in the third set were to be used in the event of human error or issues arising during sample preparation or testing that necessitated repeat testing of a sample or samples. The key steps in milk and feces sample preparation prior to application of PhMS are depicted in Figure 2.

In the case of the Milk Trial test panels, when received by each participating laboratory from NEIKER, the 10 ml milk samples were placed at 4 °C overnight and testing commenced the next morning (i.e., ~48 h after preparation by NEIKER personnel). The panel of milk samples was removed from the refrigerator and left at room temperature for 1 h to warm up. They were then placed in a waterbath (or incubator) at 35–37 °C for 10–15 min, the purpose of which was to ensure milk fat was as liquid as possible at point of centrifugation. The milk samples were centrifuged at 2,500 × g for 15 min at room temperature, after which the whey and fat fractions were discarded. The milk pellets were thoroughly resuspended in 1 ml phosphate buffered saline containing 0.05% Tween 20 (PBST). When possible, the resuspended samples were subjected to sonication at 35 KHz for 4 min in pulse mode in a sonicator bath (Ultrasonic PH 30, Fisher Scientific Ltd., or similar) to declump MAP cells present before proceeding to manual or automated PhMS with the full 1 ml sample (equivalent to 10 ml milk). This sonication treatment has been demonstrated to de-clump MAP cells without adversely impacting MAP cell viability (23). In the case of Lab 1B only, the milk pellet was resuspended in 5 ml PBS-T rather than 1 ml because a larger capacity magnetic separation instrument (PurePrep 24D, MolGen BV, The Netherlands) was being used. In the case of Lab 4, sonication treatment was applied but their sonicator bath did not have a “pulse mode.”

In the case of the Feces Trial test panels, when received by each participating laboratory from NEIKER, the tubes containing pre-weighed 1 g feces samples were placed at 4 °C overnight and testing commenced the next morning (i.e., ~ 48 h after preparation by NEIKER personnel). As shown in Figure 2, around five sterile 3 mm glass beads (supplied to each laboratory by Rapid-Myco Technologies Limited) were aseptically added to the 1 g feces samples along with 5 ml PBS pH 7.2, before being shaken and vortexed thoroughly. A low-speed centrifugation (300 × g for 5 min) was applied to sediment large particulates quickly. Then the clarified fecal supernatant (4–5 ml) was transferred to a fresh centrifuge tube and subjected to centrifugation at 2,500 × g for 10 min to pellet any MAP cells present. This cell pellet was thoroughly resuspended in 1 ml 7H9/OADC/2 mM CaCl_2_ broth before ultrasonication (as described above for milk pellet) to declump MAP cells present. The resuspended and declumped fecal pellet was subjected to manual or automated PhMS as described below.

### PhMS-qPCR testing

Participating laboratories carried out either automated PhMS using Kingfisher 1 ml, Duo Prime or Flex instruments (all Thermo Fisher, U.S.A.) or a PurePrep 24D instrument (MolGen), or manual PhMS using a suitable magnetic rack. Phage-coated beads (15 μl, supplied by Rapid-Myco Technologies) were added to 1 ml (or 5 ml in case of Lab 1B) resuspended milk pellets of 18 × 10 ml milk samples (Milk Trial Rounds 1 and 2), or 1 ml (or 5 ml in case of Lab 1B) clarified feces supernatant (prepared as described above) of 18 × 1 g feces samples (Feces Trial Rounds 1 and 2).

Alongside the milk or feces test samples, 1 ml of a 100-fold dilution of a 4–5 week old MAP culture (where available) prepared in Middlebrook 7H9/OADC/2 mM CaCl_2_ broth was tested to serve as a PhMS positive extraction control. In addition, all laboratories processed 1 ml 7H9/OADC/2 mM CaCl_2_ broth to serve as a PhMS negative extraction control. DNA was harvested after 2 h (for MAP spiked milk or feces samples) or 4 h (for naturally infected milk or feces samples) incubation of the bead suspensions at 37 °C post-PhMS by centrifugation at 10,000 × g for 2 min after a brief heat shock at 55 °C for 1 min in a dry heating block. The resulting supernatants (template DNAs) were immediately transferred to fresh tubes, and cell pellet and beads discarded. Milk-derived DNAs were tested by qPCR without clean up. Feces-derived DNAs were subjected to column purification (Zymoclean Clean and Concentrator-5 kit, Cambridge Biosciences, UK, used per manufacturer's instructions) to remove residual PCR inhibitors before qPCR. The instruction given to the participating laboratories was to proceed directly to qPCR where time permitted, but if necessary the DNA samples could be stored at −20 °C overnight prior to qPCR step.

A decision was taken prior to commencement of the inter-laboratory trials that all laboratories would use the commercially available IDEXX RealPCR MAP DNA test (Product codes: 99-56620 MAP primer mix and 99-56250 qPCR mastermix), according to the manufacturer's instructions. Therefore, before proceeding to qPCR after DNA harvesting, 1 μl of IDEXX Internal Positive Control (Product code: 99-56330, resuspended as per manufacturer's instructions) was added to each 50 μl DNA sample (including PhMS extraction controls) to monitor for any PCR inhibition. Positive and negative qPCR controls were included in each run—IDEXX positive control DNA (Product code: 99-56310) and molecular water, respectively. Duplicate qPCR reactions were run per sample, and qPCR results were interpreted as MAP positive or negative according to IDEXX RealPCR MAP DNA test instructions (IDEXX 2017)—samples with a cycle threshold (Ct) value less than 40 and a typical amplification curve were considered positive for MAP DNA, a Ct ≥40 may be suspect positive, and the absence of an amplification curve indicated a negative result. Internal positive control amplification must be observed in negative reactions to rule out inhibition of the qPCR reaction.

### Collation of inter-laboratory trial results and statistical analysis

All PhMS-qPCR results from the participating laboratories were initially reported back to Dr Iker Sevilla, NEIKER, on a results template sheet provided at the same time as the samples at each round of testing. Individual laboratory results were collated into an Excel spreadsheet before full results for each test panel were sent to Prof. Irene Grant. Statistical analysis of results was carried out using GraphPad and Microsoft Excel (with XLSTAT add-on to generate Youden plot). AusVet Epitools (24) (https://epitools.ausvet.com.au/) was used to assess Kappa agreement between PhMS-qPCR results from each laboratory and reference milk or feces culture (generated at NEIKER), and Kappa agreement between PhMS-qPCR results from different laboratories to assess test reproducibility.

Each laboratory's PhMS-qPCR results relative to reference culture results for the test samples generated at NEIKER were compiled into 2 × 2 contingency tables. Test performance characteristics [detection specificity Sp, detection sensitivity Se and trueness T (accuracy) as defined below] of the PhMS-qPCR assay applied to milk and feces were calculated using the following equations (25, 26). Table 2 explains the abbreviations used in the equations.

**Table 2:** Explanation of test result designations used in the equations to calculate PhMS-qPCR test performance characteristics.

	**Reference value positive**	**Reference value negative**
Method positive (detected)	+/+ Positive Agreement (PA)	–/+ Positive Deviation (PD)
Method negative (not detected)	+/– Negative Deviation (ND)	–/– Negative Agreement (NA)

Sensitivity (Se), is the percentage of positives correctly identified from the total of true positive test portions (MAP spiked or MAP positive naturally infected samples), which was estimated as:


$$\begin{array}{c} \frac{\text{(PA)}}{\left(\text{PA}+\text{ND}\right)}\times 100 \end{array}$$ (1)

Specificity (Sp), is the percentage of negatives correctly identified from the total of true negative test portions (not spiked or not naturally infected samples), which was estimated as:


$$\begin{array}{c} \frac{\text{NA}}{\left(\text{PD}+\text{NA}\right)}\times 100 \end{array}$$ (2)

Trueness (T), is the percentage of results (including positive and negative) correctly assigned considering the reference value, which was estimated as:


$$\frac{\text{(PA+NA})}{\left(\text{PA }+\text{ ND }+\text{ PD }+\text{ NA}\right)}\times 100$$ (3)

Similarly, 2 × 2 contingency tables were compiled of each laboratory's PhMS-qPCR results relative to every other participating laboratory's PhMS-qPCR results to assess reproducibility by McNemar's Chi square test when testing articificially spiked milk (spiked raw and UHT milk) and naturally infected feces.

## Results

### Milk inter-laboratory trial

**Figure 3:**
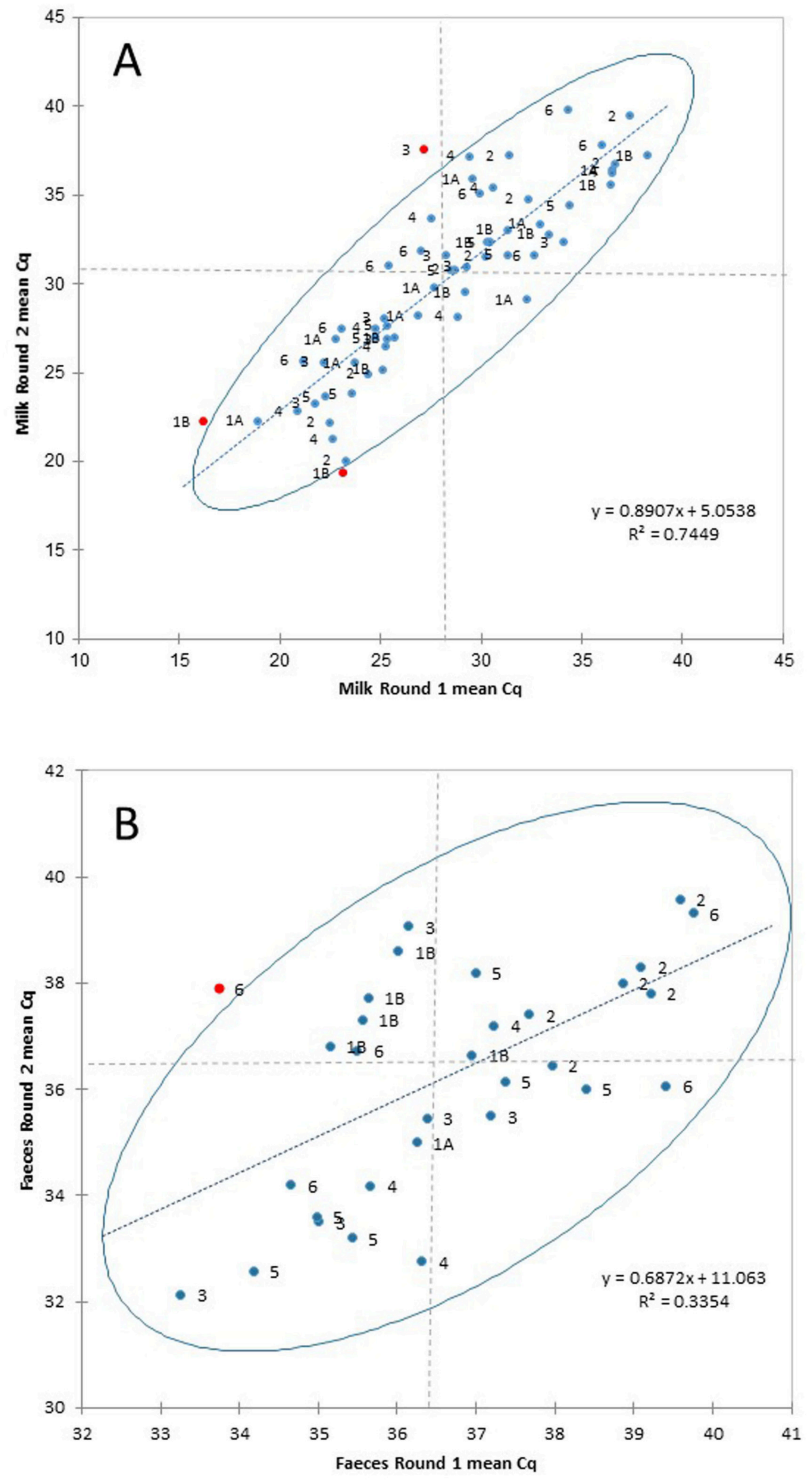
Mean PhMS-qPCR Cq results (of duplicate qPCR reactions) reported by the six participating laboratories using seven PhMS-qPCR approaches (1A, 1B, 2, 3, 4, 5, and 6) for **(A)** UHT and raw milk samples spiked with laboratory-grown MAP (at concentrations from 10^1^ to 10^5^ cfu/ml) and **(B)** naturally MAP-infected feces tested, during Rounds 1 and 2 of the Milk and Feces Trials. The results are plotted as a Youden plot with a 95% confidence ellipse around the results. Red circles highlight outlier results. Dashed lines indicate medians of Round 1 and Round 2 Cq data in each case.

Table 3 summarizes the results of the milk trial to evaluate detection of viable MAP by PhMS-qPCR in spiked raw and UHT milk, combining results obtained for UHT and raw milk by each lab because there was no significant difference in the results obtained for the two types of milk and the overall number of spiked milk samples tested was low (*n* = 12 per round). Table 3 also indicates how PhMS was performed in each of the laboratories, i.e., manually (two laboratories) or using an automated magnetic separation instrument (four labs by five methods). During Round 1, five of the seven PhMS-qPCR methods used in the six laboratories correctly detected viable MAP in all five spiked UHT milk samples, whereas in the case of spiked raw milk samples four of the seven PhMS-qPCR methods successfully detected viable MAP in all five spiked samples. A stepwise increase in PhMS-qPCR Cq values for decreasing spiked MAP levels was observed in most cases, although actual Cq values returned for the same milk samples with IDEXX RealPCR MAP DNA kit varied between labs. A Youden plot was generated to compare Cq values obtained in Milk Trial Rounds 1 and 2 by the different PhMS-qPCR approaches in the various laboratories (Figure 3A). There was good correlation (*r*^2^ = 0.7449) between the Round 1 and 2 results, and all but three of the data points fell within the 95% confidence ellipse; two Lab 1B results and one Lab 3 result were outliers. A small number of apparent false positive results for non-spiked milks were encountered in some labs (Lab 1B for both types of milk, Lab 5 for raw milk and Lab 6 for UHT milk; Table 3).

**Table 3:** Summary of inter-laboratory trial results for spiked UHT and raw milk (*n* = 12) showing calculated Kappa agreement between PhMS-qPCR assay and culture results (^*^p < 0.05, ^**^ *p* < 0.01, ^***^ *p* < 0.001, ^NS^ not significant) and estimates of PhMS-qPCR test characteristics (detection specificity, detection sensitivity, and trueness). Expected results (both rounds): 10, 0, 0, 2.

**Lab ID**	**Type of magnetic separation (Instrument)**	**+/+ (PA)**	**+/– (ND)**	**–/+ (PD)**	**–/– (NA)**	**Kappa (95% CI)**	***p* Value**	**Detection specificity (Sp)**	**Detection sensitivity (Se)**	**Trueness (T)**
**Round 1**										
1A	Automated (Kingfisher 1 ml)	10	0	0	2	1.000 (1.000, 1.000)	0.0003^***^	100	100	100
1B	Automated (PurePrep 24D)	10	0	2	0	–		0	100	83.3
2	Automated (Kingfisher Flex)	10	0	0	2	1.000 (1.000, 1.000)	0.0003^***^	100	100	100
3	Automated (Kingfisher Flex)	8	2	0	2	0.5714 (0.0815, 1.0613)	0.0142^*^	100	80	83.3
4	Automated (Kingfisher DUO Prime)	9	1	0	2	0.7500 (0.2958, 1.2042)	0.0036^**^	100	90	91.7
5	Manual	8	2	1	1	0.2500 (−0.3716, 0.8716)	0.1855^NS^	50	80	75
6	Manual	10	0	1	1	0.6250 (−0.0273, 1.2773)	0.0098^**^	50	100	91.7
All	Various	65	5	4	10	0.6250 (0.4016, 0.8484)	0.0000^***^	71.4	92.9	89.3
**Round 2**										
1A	Automated (Kingfisher 1 ml)	10	0	1	1	0.6250 (−0.027, 1.2713)	0.0098^**^	50	100	91.7
1B	Automated (PurePrep 24D)	10	0	2	0	–		0	100	83.3
2	Automated (Kingfisher Flex)	9	1	0	2	0.7500 (0.2958, 1.2042)	0.0036^**^	100	90	91.7
3	Automated (Kingfisher Flex)	8	2	0	2	0.5714 (0.0815, 1.0613)	0.0142^*^	100	80	83.3
4	Automated (Kingfisher DUO Prime)	9	1	0	2	0.7500 (0.2958, 1.0613)	0.0036^**^	100	90	91.7
5	Manual	10	0	2	0	–		0	100	83.3
6	Manual	9	1	0	2	0.7500 (0.2958, 1.0613)	0.0036^**^	100	90	91.7
All	Various	65	5	5	9	0.5714 (0.3339, 0.8089)	0.0000^***^	64.3	93.3	88.1

PA, positive agreement; ND, negative deviation; PD, positive deviation; NA, negative agreement; Sp, detection specificity; Se, detection sensitivity; T, trueness.

A full limit of detection (LOD) experiment was not carried out, but an indication of the LOD of the PhMS-qPCR applied to milk was obtained by considering the lowest MAP spiking level at which most laboratories correctly detected viable MAP (20). The lowest MAP concentration tested differed between rounds 1 and 2; estimated to be 60 and 300 viable MAP/ml of milk, respectively, based on a colony count of the MAP ATCC 19698 inoculum used to spike the milks. Four of the seven PhMS-qPCR protocols yielded a positive result for raw milk at the lowest spiking level in both rounds, and 4 of 7, and 5 of 7 PhMS-qPCR protocols yielded a positive result for spiked UHT milk at the lowest spiking level in rounds 1 and 2, respectively (Table 4). The LOD of the PhMS-qPCR assay is, therefore, estimated to be 10–100 viable MAP/ml of milk.

**Table 4:** Limit of detection (LOD) of the PhMS-qPCR assay applied to test MAP-spiked raw and UHT milk.

**Lab ID**	**Round 1 spiking levels (viable MAP/ml milk)**						**Round 2 spiking levels (viable MAP/ml milk)**					
	**6** × **10**^5^	**6** × **10**^4^	**6** × **1 0**^3^	**6** × **10**^2^	**6** × **10**^1^	**Non-spiked**	**3** × **10**^6^	**3** × **10**^5^	**3** × **10**^4^	**3** × **10**^3^	**3** × **10**^2^	**Non-spiked**
**Raw milk** ^*^												
1A	P	P	P	P	P	NA	P	P	P	P	P	NA
1B	P	P	P	P	P	P^**^	P	P	P	P	P	P^**^
2	P	P	P	P	P	NA	P	P	P	P	P	NA
3	P	P	P	P	NA	NA	P	P	P	P	NA	NA
4	P	P	P	P	NA	NA	P	P	P	P	NA	NA
5	P	P	P	P	NA	P^**^	P	P	P	P	P	P^**^
6	P	P	P	P	P	NA	P	P	P	P	NA	NA
Frequency of detection	7/7	7/7	7/7	7/7	4/7	2/7	7/7	7/7	7/7	7/7	4/7	2/7
**UHT milk**												
1A	P	P	P	P	P	NA	P	P	P	P	P	P^**^
1B	P	P	P	P	P	P^**^	P	P	P	P	P	P^**^
2	P	P	P	P	P	NA	P	P	P	P	NA	NA
3	P	P	P	P	NA	NA	P	P	P	P	NA	NA
4	P	P	P	P	P	NA	P	P	P	P	P	NA
5	P	P	P	P	NA	NA	P	P	P	P	P	P^**^
6	P	P	P	P	S	S	P	P	P	P	P	NA
Frequency of detection	7/7	7/7	7/7	7/7	4/7	1/7	7/7	7/7	7/7	7/7	5/7	3/7

Viable MAP spiking levels in the milk were estimated by plating the MAP inoculum for Rounds 1 and 2 to obtain colony counts. PhMS-qPCR results were interpreted per IDEXX RealPCR MAP DNA kit instruction manual: P, positive (Cq <40), S, suspect (Cq 40 or above). NA, no amplification/negative. ^*^Milk obtained from single confirmed Johne's disease negative cow. ^**^Apparent false positive result for non-spiked milk.

Reproducibility of the PhMS-qPCR assay for testing milk in different laboratories was assessed by calculating Cohen's Kappa agreement between each laboratory's results and those of all other laboratories (Supplementary Table S6). A Kappa value 0.6 or above (“moderate” or better agreement) (27) was considered to indicate acceptable reproducibility of the assay between laboratories. Lab 1B results, for which calculated Cohen's Kappa values of 0.250–0.428 for raw milk and 0.3077–0.4286 for UHT milk) were obtained, did not show good agreement with most other laboratories. Kappa values close to or above 0.60 (ranging from 0.5714 to 1.000) were obtained in all other cases.

Detection Se and Sp values and T for the PhMS-qPCR assay applied to spiked milk were calculated from each laboratory's results relative to NEIKER's categorization of samples in each panel as MAP culture positive or negative, using Equations 1–3 provided above, and then overall test characteristics were calculated based on milk results from all laboratories (Table 3). Results for the two rounds of the milk trial were very similar, the only difference being an extra false positive (positive deviation) in Lab 5, which impacted detection Sp calculation for Round 2 (64.3% instead of 71.4%; Table 3). Four of the seven PhMS-qPCR protocols achieved 100% Sp in both rounds. However, there seemed to be some problem with method 1B for both rounds, and method 5 in Round 2, as calculated Sp was zero (based on results of just two non-spiked samples). Detection Se and T values were consistently good for PhMS-qPCR applied to milk for all labs, at 93.1 and 88.7% overall, respectively.

When six naturally infected raw milks were tested during Round 1 of the Milk Trial there were no PhMS-qPCR positive results at all. However, six PhMS-qPCR positive results were recorded out of 42 results across all laboratories/methods during Round 2 (Cow 1 positive in Labs 1B and 5, Cow 3 positive in Lab 1B, Cow 4 positive in Lab 1B, and Cow 6 positive in Labs 1B and 5; Supplementary Table S2). The positive PhMS-qPCR results were mostly obtained by Lab 1B who performed PhMS using the PurePrep 24D instrument (MolGen Limited) which can process a larger sample volume (milk pellet was resuspended in 5 ml rather than 1 ml). These PhMS-qPCR results were not in agreement with culture results obtained by NEIKER for the six naturally infected milk samples but were more similar to the qPCR results they obtained (Supplementary Table S2). These results could potentially indicate that the PhMS-qPCR assay has greater detection capability for viable MAP than HPC decontamination and culture on HEYM. Due to the very inconsistent detection of viable MAP in the naturally infected milk samples tested, results were not included in Se, Sp or T calculations.

### Feces inter-laboratory trial

Table 5 summarizes the results obtained by each laboratory during the Feces Trial to evaluate detection of viable MAP by PhMS-qPCR in naturally infected feces. During Round 1, all laboratories had trouble detecting viable MAP in the spiked feces samples that formed part of the test panel (Figure 1B), and generally only the samples containing the highest concentrations of spiked MAP tested PhMS-qPCR positive. Supplementary Table 3A shows that the presence of viable MAP was inconsistently detected by two laboratories (Rapid-Myco and FLI) across the MAP spiking concentrations when the replicate sets of feces samples were cultured in parallel to PhMS-qPCR, and in a third laboratory (NEIKER) viable MAP was not confirmed at all due to contamination issues. The other 12 samples in the Round 1 test panel were confirmed MAP-infected feces (*n* = 9) or MAP negative feces (*n* = 3). Parallel HEYM culture confirmed the presence of viable MAP in all but one of the feces that had been designated as MAP positive (sample N9, which had corresponding Cq values of ≥35 by the two PCR methods applied; Supplementary Table 3B). Table 5 shows that there were a lot of false negative results (negative deviation) during Round 1 for the naturally MAP-infected feces samples, and the detection capabilities of three PhMS-qPCR methods applied in two laboratories (1A, 1B, and 4) were noticeably lower than the four other methods. There was “fair agreement” (26) between PhMS-qPCR and parallel culture results for the naturally infected feces tested during Round 1; kappa value was 0.3895 (95% CI 0.2273, 0.5517, *p* = 0.000) on the basis of combined results from all laboratories, ranging from 0.111 to 0.6364 for individual laboratories. A modified Lab 1A method (method 1A^*^ in Table 5) which involved testing the full 5 ml of clarified fecal supernatant (equivalent to 1 g of feces) rather than 1 ml (equivalent of 0.2 g of feces), by introduction of an additional centrifugation step, performed much better—only one false negative result resulting in calculated Kappa agreement with reference result of 0.800 (*p* value 0.0023). Therefore, it was decided that the test panel for feces trial Round 2 would consist entirely of confirmed naturally MAP-infected feces (*n* = 9) or MAP negative feces (*n* = 9), and the amended sample preparation protocol (as just described) would be performed in all laboratories. This protocol change more than halved the number of false negative results (10 instead of 26, Table 5) and Kappa agreement with reference results doubled to 0.7780 (“substantial agreement,” 28). During Round 2, with the exception of method 1A^*^ (Kappa 0.2220, 95% CI −0.2254, 0.6699, *p* = 0.17), all PhMS-qPCR methods performed well (Kappa values 0.7780–1.000, *p* < 0.001 in all other cases). A Youden plot was generated to compare Cq values obtained in Feces Trial Rounds 1 and 2 by the different PhMS-qPCR approaches in the various laboratories for the same naturally MAP infected feces samples (Figure 3B). There was reasonable correlation (*r*^2^ = 0.3354) between the Round 1 and 2 results and all but one of the data points fell within the 95% confidence ellipse, with one Lab 6 result an outlier. A broader spread of datapoints (greater variation in PhMS-qPCR Cq results) was observed for naturally infected feces results (Figure 3B) than for spiked milk results (Figure 3A).

**Table 5:** Summary of inter-laboratory trial results for naturally infected feces showing calculated Kappa agreement between PhMS-qPCR assay and culture results (^*^p < 0.05, ^**^p < 0.01, ^***^p < 0.001, ^NS^ not significant) and estimates of PhMS-qPCR test characteristics (detection specificity, detection sensitivity, and trueness).

**Lab ID**	**Type of magnetic separation (Instrument)**	**+/+ (PA)**	**+/– (ND)**	**–/+ (PD)**	**–/– (NA)**	**Kappa (95% CI)**	***p* Value**	**Sp**	**Se**	***T***
**Round 1**										
1A	Automated (Kingfisher 1 ml)	2	7	0	3	0.1250 (−0.0776, 0.3276)	0.1855^NS^	66.7	22.2	41.7
1A	Automated (Kingfisher 1 ml)^a^	8	1	0	3	0.8000 (0.4323, 1.1677)	0.0023^**^	100.0	88.8	91.7
1B	Automated (PurePrep 24D^b^)	7	2	2	1	0.1110 (−0.4855, 0.7078)	0.3502^NS^	33.3	77.7	66.7
2	Automated (Kingfisher Flex)	7	2	0	3	0.6364 (0.2708, 1.0649)	0.0090^**^	100.0	77.7	83.3
3	Automated (Kingfisher Flex)	5	4	0	3	0.3846 (−0.0035, 0.7729)	0.0455^*^	100.0	55.5	66.7
4	Automated (Kingfisher DUO Prime)	3	6	0	3	0.2000 (−0.0716, 0.4716)	0.1241^NS^	100.0	33.3	50.0
5	Manual	7	2	0	3	0.6364 (0.2708, 1.0649)	0.0090^**^	100.0	77.7	83.3
6	Manual	7	2	1	2	0.4935 (−0.1544, 0.9544)	0.0355^*^	66.7	77.7	75.0
All	Various	46	26	3	21	0.3895 (0.2273, 0.5517)	0.0000^***^	87.5	63.9	69.8
**Round 2**										
1A	Automated (Kingfisher 1 ml)^a^	5	4	3	6	0.2220 (−0.2254, 0.6699)	0.171^NS^	66.7	55.6	61.1
1B	Automated (PurePrep 24D)	8	1	0	9	0.8890 (0.6786, 1.0992)	0.0001^***^	100.0	88.8	94.4
2	Automated (Kingfisher Flex)^a^	8	1	0	9	0.8890 (0.6786, 1.0992)	0.0001^***^	100.0	88.8	94.4
3	Automated (Kingfisher Flex)^a^	7	2	0	9	0.7780 (0.4947, 1.0609)	0.0004^***^	100.0	77.7	88.8
4	Automated (Kingfisher DUO Prime)^a^	8	1	0	9	0.8890 (0.6786, 1.0992)	0.0001^***^	100.0	88.8	94.4
5	Manual^a^	9	0	0	9	1.000 (1.000, 1.000)	0.0000^***^	100.0	100.0	100.0
6	Manual^a^	8	1	1	8	0.7780 (0.4947, 1.0609)	0.0005^***^	88.8	88.8	88.8
All	Various	53	10	4	59	0.7780 (0.4947, 1.0609)	0.0000^**^	93.7	84.1	88.9

Expected results: Round 1-9, 0, 0, 3 and Round 2-9, 0, 0, 9. PA, positive agreement; ND, negative deviation; PD, positive deviation; NA, negative agreement; Sp, detection specificity; Se, detection sensitivity; T, trueness. ^a^Extra centrifugation step added to feces sample preparation to enable testing of full 5 ml clarified fecal supernatant (equivalent of whole 1 g feces) rather than just 1 ml (equivalent of 0.2 g feces). ^b^Larger volume magnetic separation instrument that permitted testing of 5 ml clarified fecal supernatant directly.

Reproducibility of the PhMS-qPCR assay applied to feces in the different laboratories was assessed by calculating Cohen's Kappa agreement between each laboratory's results and those of all other laboratories (Supplementary Tables S7A, B). Results for feces trial Rounds 1 and 2 were analyzed separately due to the change in sample preparation protocol before PhMS in all laboratories between rounds. For Round 1, not many of the laboratory comparisons yielded a Cohen's Kappa value 0.6 or above (Labs 2 and 3 vs. other laboratories mainly). Once again Lab 1B results showed particularly poor agreement with other laboratories (negative Cohen's Kappa values −0.0909 to −0.3333), and Lab 1A also showed poorer but at least positive agreement with other laboratories (Kappa values 0.0625 to 0.3333; Supplementary Table S7A). With the change in feces sample preparation during Round 2 reproducibility figures substantially improved; Cohen's Kappa values ranging from 0.5556 to 0.8889 were obtained for all comparisons with the exception of Lab 1A (0.1000 to 0.3250, Supplementary Table S7B).

Results of testing naturally infected feces (Table 5) were used to calculate estimates of PhMS-qPCR test performance characteristics when applied to feces. During Round 1 of the feces trial there were a lot of false negative results (negative deviation), which adversely impacted the detection Se values calculated −65.3% overall, range 22.2%−77.7%. During Round 2, all laboratories employed the modified feces sample preparation protocol (i.e., inclusion of a second centrifugation step on the 5 ml clarified fecal supernatant and resuspension of cell pellet in 1 ml 7H9/OADC/2 mM CaCl_2_ broth) prior to PhMS. Calculated test characteristics for the PhMS-qPCR assay applied to feces improved substantially from Round 1 to Round 2 across the board—detection Sp 87.5%−93.7%, detection Se 63.9%−84.1%, and T 69.8%−88.9%, respectively.

### Discussion

The PhMS-qPCR assay was developed to be a rapid alternative to (HPC decontamination and) culture for detecting viable MAP in milk and feces (12, 19), providing results within 24 h of testing a sample rather than having to wait weeks for colonies to appear on a HEYM slant or growth to be detected in a MGIT tube (3). MAP-specific qPCR is the endpoint detection step of the assay, however only DNA from viable MAP is released by phage action during the incubation period after PhMS. Dead MAP cells will not permit phage amplification so phage enzymes are not produced to cause cell lysis and release of DNA. This is an important point for readers to note, as the comparator reference test for PhMS-qPCR is culture, not direct qPCR, when calculating test performance characteristics. NEIKER performed direct qPCR on feces and raw milk samples in order to identify MAP-infected cattle from which feces and milk could be sourced and to verify the presence and relative quantities of MAP after spiking. Certain other laboratories performed direct qPCR and HPC decontamination and HEYM culture on test panels to further verify NEIKER's categorization of samples as MAP infected/spiked or not.

Ring trials (otherwise termed inter-laboratory test comparisons) are useful exercises to carry out during test development and validation to facilitate comparison of the detection capability and consistency of detection (termed reproducibility) of a new diagnostic test (21). In technology transfer, an established method is usually used in a different laboratory, by different analysts, or with different instrumentation (28). Successful technology transfer is an important consideration during validation of any new diagnostic test, since a test that performs well in the originator laboratory but then does not perform as well in potential adopter laboratories is not going to be a useful test. Moreover, assessment of reproducibility constitutes Stage 3 of validation of a new veterinary diagnostic test, as set out by the WOAH (22). In this study, six laboratories across Europe performed the PhMS-qPCR protocol with common reagents (phage-coated paramagnetic beads and bead resuspension broth supplied by Rapid-Myco Technologies Limited and IDEXX RealPCR MAP DNA kits) on aliquots of identical MAP-spiked or naturally infected milk or feces samples (prepared centrally at NEIKER before distribution to other participating laboratories), using whatever existing equipment each laboratory had access to (Supplementary Table S5). Thus, the study meets the ideal requirements of an experiment to assess reproducibility of a test stipulated by OIE (now WOAH), i.e., at least three laboratories should test the same panel of samples (blinded) using the same protocol, reagents and controls (20).

The WOAH validation pathway for a new veterinary diagnostic test (22) recommends that the composition of test panels should reflect the inherent biological variability of the target pathogen (different serotypes) and circulating or geographically relevant strains. Furthermore, where possible, the samples should be prepared from known positive field samples (in our case naturally contaminated MAP-positive milk and feces) or from positive samples spiked into suitable matrix to mimic a true test sample (e.g., negative bovine milk or feces artificially spiked with a known concentration of MAP bacteria). The WOAH recommendation is that the test panel should contain a full representation of concentrations covering the operating range of the assay in animals of the target population, to ensure evidence of reproducibility at a range of possible analyte levels, not just at either end of the scale, i.e., strong-positive and weak-positive (20, 22). When deciding on the size and composition of the test panels to be sent to participating laboratories in this study (outlined in Figure 1), our aim was to include both artificially MAP-spiked and naturally MAP-infected milk or feces samples. Our test panel size of 18 samples in total for both trials almost meets WOAH guidance that evaluation panels should contain 20 or more samples (29). Duplication in the milk trial was achieved by sending identical test panels on two separate occasions to participating laboratories, rather than duplicate samples within each panel. Due to issues with all laboratories having trouble detecting viable MAP in the spiked feces samples during Round 1 of the feces trial, the decision was taken to change the composition of the test panel for the second round. For Round 2, equal numbers of known MAP positive and MAP negative naturally infected feces samples were included and no MAP-spiked feces at all. This change permitted a more reliable determination of detection Sp and Se of the PhMS-qPCR assay applied to feces using Round 2 results. It is unclear why viable MAP were not detectable in some of the spiked feces 2–3 days post-spiking (perhaps loss of viability of laboratory-grown MAP when exposed to harsh feces environment for 2 days between spiking and testing in the various laboratories), but this difficulty had been encountered previously by Rapid-Myco Technologies staff during PhMS-qPCR test optimisation when spiked feces are tested (unpublished data).

Reproducibility testing must be carried out when validating any new detection method as it permits assessment of the robustness of a protocol when undergoing technology transfer to other laboratories (20, 22). Ideally, the different laboratories involved in inter-laboratory trials should use identical protocols, reagents and equipment, but in reality this is seldom the situation; as was the case during the milk and feces PhMS-qPCR trials. Whilst the same reagents (phage-coated beads and bead resuspension broth) and qPCR kit (IDEXX RealPCR MAP DNA kit) were used in all participating laboratories, Supplementary Table S5 clearly shows the variety of different equipment (sonicator bath, magnetic separation device, qPCR instrument) employed to perform the PhMS-qPCR assay across the laboratories. Waugh and Clark (20) discussed multiple factors that may affect veterinary diagnostic test reproducibility and summarized the common sources of variability as laboratory environment, technician capability, calibration of instruments, and consistency and quality of assay reagents. Cohen's kappa agreement between results obtained during the milk and the feces inter-laboratory trials were calculated to evaluate PhMS-qPCR assay capability. In the case of the milk trial, reproducibility of the PhMS-qPCR assay between laboratories for the spiked raw and UHT milk samples was generally good (Cohen's Kappa values 0.5714–1.000 [moderate to very good agreement (30)] for all comparisons except for Lab 1B, Supplementary Table S6). In the case of the feces trial, reproducibility of the PhMS-qPCR assay was very variable during Round 1 (Cohen's Kappa values ranging from −0.3333 to 1.0000, “less agreement than expected by chance” to “very good” agreement (30), with Lab 1B results once again being particularly divergent [Cohen's Kappa values −0.3333 to −0.0909, “worse agreement than expected by chance” (30), Supplementary Table S7A]. However, during Round 2 there was substantial improvement in test reproducibility [Cohen's Kappa values 0.5556 to 0.8889, “moderate” to “very good” agreement (30)], except for Lab 1A comparisons [Cohen's Kappa values 0.1000 to 0.3250, “poor” to “fair” agreement (30), Supplementary Table S7B]. A PurePrep 24D automated magnetic separation instrument was employed in Lab 1B which can process a 5 ml sample directly, whereas the other laboratories were able to test a 1 ml sample volume maximum (1 ml resuspended milk pellet during milk trial, 1 ml sub-sample of 5 ml clarified fecal supernatant during feces trial round 1, or 1 ml of centrifuged 5 ml clarified fecal supernatant during feces trial round 2). It makes sense that if more (all) of a prepared sample can be tested then the chances of successfully detecting the target pathogen, if present, will increase. This probably explains why Lab 1B results were not in agreement with other laboratories until Round 2 of the feces trial when all laboratories tested the full 5 ml clarified feces supernatant; Lab 1B had been detecting more of the naturally MAP-infected feces samples correctly in Round 1 and other protocols had been missing MAP positive samples. The reason why Lab 1A results did not agree with all other laboratories during Round 2 of the feces trial is unclear. Anecdotally, the Kingfisher 1mL instrument employed in this laboratory has since stopped working after many years of use for PMS and PhMS, so malfunction of the mixing and/or magnetic separation capabilities of the instrument may have been at play at the time of feces trial Round 2.

A recent review by Martins et al. (18) mentions phage-based methods as a new diagnostic for JD, but the authors highlighted that there was a knowledge gap in relation to their test characteristics when applied to feces and blood. Results of the inter-laboratory trials reported here permitted estimation of the performance characteristics of the new PhMS-qPCR assay for detection of viable MAP in milk and feces. An inter-laboratory trial to test blood was not logistically possible due to a requirement for fresh blood to extract the buffy coat fraction from when testing this sample matrix by PhMS-qPCR (19, 32). Tables 3, 5 provide a complete summary of the calculated test characteristics of the PhMS-qPCR assay, relative to reference culture results for milk and feces generated by NEIKER, respectively. The calculated test performance characteristics (detection Sp 67.9%, detection Se 93.1% and T 88.7% for milk and detection Sp 93.7%, detection Se 84.1% and T 88.9% for feces) contrast sharply with results for an earlier iteration of the present PhMS-qPCR assay, the PMS-phage assay (31), previously evaluated by Butot et al. (25). The PMS-phage assay was a much more cumbersome test to apply (requiring molten agar and a *Mycobacterium smegmatis* culture for a plaque assay) and took longer to yield results (2–3 days) than the current PhMS-qPCR assay [ <24 h; see Grant review (5) for more detail of the differing principles of the two tests]. Butot et al. (25) reported that the PMS-phage assay performed poorly relative to MAP culture and qPCR when applied to test spiked milk. The detection Se of the PMS-phage assay was only 40%, whereas culture and qPCR had detection Se of 83% and 76–94% (dependent on qPCR target, f57 or IS*900*), respectively, for testing MAP-spiked milk. Trueness of the PMS-phage assay was 49%, whereas culture and qPCR applied to MAP-spiked milk had T values of 89 and 93%, respectively. In contrast, during the present trials the PhMS-qPCR assay proved easy to apply in the participating laboratories and generally yielded good results during both trials (Tables 3, 5), even though this was only the first few times the majority of laboratory personnel involved had ever applied the PhMS-qPCR test. A range of different magnetic separation instruments were employed in the various laboratories, but how PhMS was performed (manually or using an automated instrument) did not appear to impact calculated test characteristics.

The sample preparation protocols for milk and feces in advance of PhMS (outlined in Figure 2) had been carefully optimized by Rapid-Myco Technologies personnel prior to the inter-laboratory trials taking place. Considering PhMS-qPCR results obtained, including no evidence of non-amplification of the IDEXX IPC during qPCR, it seems that only minor tweaks to the original feces sample preparation protocol are necessary going forward to maximize the detection Se of the PhMS-qPCR assay; the main one being the additional centrifugation step for the clarified fecal supernatant to permit processing the entire 1 g test feces sample through PhMS rather than one fifth of a 1 g sample. Rapid-Myco Technologies Ltd. do not envisage any further sample preparation or PhMS-qPCR optimization is going to be needed. The estimated LOD of the PhMS-qPCR assay based on the spiked milk results is in the range 10–100 viable MAP/ml milk, which is higher than LOD_50%_ determined by Foddai and Grant (12) when the assay was originally being developed −10.0 MAP cells/50 ml milk (95% CI 1.20–82.83). The milk sample preparation protocol prior to PhMS does not appear to need to be altered substantially considering results obtained. Laboratories probably just need more practice with unfamiliar milk preparation steps (pre-warming before centrifugation and inclusion of sonication to declump MAP cells present). Also, increased sensitivity for detecting viable MAP in milk will likely only be achieved in future by testing larger volumes of milk (50 ml rather than 10 ml tested here), given that lower numbers of MAP (closer to the minimum detection limit of the PhMS-qPCR assay) are shed in milk of infected cattle compared to feces. The capability of the PhMS-qPCR assay to detect viable MAP in naturally infected milk has been demonstrated previously by Foddai et al. (13) who tested 15–40 ml volumes of bulk tank milk and 50 ml milk from individual cattle, and not just a 10 ml volume. Our recommendation going forward would be to test 50 ml milk where possible, in order to maximize detection sensitivity of the PhMS-qPCR assay.

The limitations of the study mostly relate to the size and composition of the test panels. In hindsight, a higher number of non-spiked raw and UHT milks should have been included in the test panels for the milk trial, so that estimates of Sp were not based on results of just two non-spiked milk samples per laboratory. Also, the inclusion of six “naturally infected” milk samples that had not been conclusively demonstrated to have any viable MAP present at NEIKER by culture probably led to wasted effort, as the results generated for these samples (Supplementary Table S2) could not be analyzed in any sensible fashion. Ideally, the two test panels for the feces trial would have remained the same for the two rounds of testing. However, due to problems with recovery of viable MAP from feces spiked with laboratory-grown MAP in Round 1 it was considered more appropriate to test equal and higher numbers of known MAP-infected and non-infected feces during Round 2, so that better estimates of detection Se and Sp could be calculated. This same issue has also prevented us from determining an LOD for the PhMS-qPCR assay applied to spiked bovine feces to date.

## Conclusion

The results of the inter-laboratory milk and feces trials described herein demonstrate that the novel phage-based PhMS-qPCR assay to quickly detect viable MAP in milk or feces was reproducible outside the progenitor laboratory in five different laboratories across Europe. Estimates of performance characteristics of the assay applied to milk (detection Sp 67.9%, detection Se 93.1% and T 88.7%) and feces (detection Sp 93.7%, detection Se 84.1% and T 88.9%) were generally very acceptable for a potential diagnostic test. Hence, the PhMS-qPCR phage-based assay shows considerable promise as a rapid test for detecting viable MAP in these two veterinary specimen types at least. It doesn't require expensive magnetic separation equipment as PhMS can be performed manually if necessary, the phage-coated magnetic beads are a low cost reagent (a few £ or $ per sample), and the number of samples that can be processed in a day is only limited by the capacity of available centrifuges. Further and fuller validation of the assay for testing milk, feces and other specimen types (blood and environmental samples) is ongoing. A report on first application of the PhMS-qPCR assay to test for viable MAP and *Mycobacterium bovis* in blood of Northern Ireland cattle has just been published (32).

## Data Availability

The original contributions presented in the study are included in the article/Supplementary material, further inquiries can be directed to the corresponding author.
