# Supplementary material for: Evaluation of the reproducibility and performance characteristics of the Phagomagnetic separation-qPCR assay for rapidly detecting viable Mycobacterium avium subsp. paratuberculosis in bovine milk and feces
Source: Front Vet Sci. 2025 Dec 15;12:1677096. doi: 10.3389/fvets.2025.1677096 (PMC12746688; doi:10.3389/fvets.2025.1677096)
Supplement: Supplementary file 1 [file Table_1.docx]

**Supplementary Information**

**Evaluation of the Reproducibility and Performance Characteristics of the Phagomagnetic Separation (PhMS)-qPCR Assay for Rapidly Detecting Viable *Mycobacterium avium* subsp. *paratuberculosis* in Bovine Milk and Faeces**

Irene R. Grant^1,2^, Iker A. Sevilla^3^, Elena Molina^3^, Beatriz Romero Martinez^4,5^, Víctor Lorente-Leal^4,6^, Virginie C. Thibault-Poisson^7,8^, Martina Cechova^9^, Heike Köhler^10^

^1^ School of Biological Sciences, Queen’s University Belfast, 19 Chlorine Gardens, Belfast BT9 5DL, United Kingdom

^2^ Rapid-Myco Technologies Limited, 63 University Road, Belfast BT7 1NF, United Kingdom

^3^ Departamento de Sanidad Animal, NEIKER-Instituto Vasco de Investigación y Desarrollo Agrario, Basque Research and Technology Alliance (BRTA), Berreaga 1, Derio, Bizkaia, 48160, Spain

^4^ Centro de Vigilancia Sanitaria Veterinaria (VISAVET), Universidad Complutense de Madrid, Madrid, Spain

^5^ Departamento de Sanidad Animal, Facultad de Veterinaria, Universidad Complutense de Madrid, Madrid, Spain

^6^ Departamento de Genética, Fisiología y Microbiología, Facultad de Ciencias Biológicas, Universidad Complutense de Madrid, Madrid, Spain

^7^ ANSES, Ploufragan-Plouzané-Niort Laboratory, Ruminant Diseases and Welfare Unit, Niort, France

^8^ GDS France (National Animal Health Farmers’ Organization), Paris, France

^9^ Department of Microbiology and Antimicrobial Resistance, Veterinary Research Institute, Brno, Czech Republic

^10^ Institute of Molecular Pathogenesis, Friedrich-Loeffler Institut, Jena, Germany

**Corresponding author:** Prof Irene Grant, Queen’s University Belfast, [i.grant@qub.ac.uk](mailto:i.grant@qub.ac.uk)

**TABLE 1** Confirmation of MAP levels in MAP-spiked raw and UHT milks at NEIKER. Presence or absence of viable MAP or MAP DNA was assessed by culture on M7H9-OADC-MJ agar and Herrold’s egg yolk ANV slants (HEYM), and by Adiapure DNA extraction and IDEXX RealPCR MAP DNA test, respectively, for the two rounds of PhMS-qPCR testing.

| **Target spiking level (MAP cfu/ml milk)** | **ROUND 1** | | | | **ROUND 2** | | | |
| --- | --- | --- | --- | --- | --- | --- | --- | --- |
|  | **Estimated no. of viable MAP in spiked milks (CFU/ml)*** | **HEYM culture** of spiked milks** | **Adiapure DNA extraction of spiked milks + IDEXX qPCR** | | **Estimated no. of viable MAP in spiked milks (CFU/ml)*** | **HEYM culture*of spiked milks** | **Adiapure DNA extraction of spiked milks + IDEXX qPCR** | |
|  |  |  | **Mean Cq** | **Result***** |  |  | **Mean Cq** | **Result**** |
| **UHT milk** |  |  |  |  |  |  |  |  |
| 10^5^-10^6^ | 6x10^5^ | P | 15.29 | P | 3x10^6^ | P | 16.41 | P |
| 10^4^-10^5^ | 6x10^4^ | P | 18.19 | P | 3x10^5^ | P | 18.51 | P |
| 10^3^-10^4^ | 6x10^3^ | P | 21.67 | P | 3x10^4^ | P | 21.88 | P |
| 10^2^-10^3^ | 6x10^2^ | P | 30.48 | P | 3x10^3^ | P | 24.84 | P |
| 10^1^-10^2^ | 6x10^1^ | N | 36.20 | P | 3x10^2^ | P | 34.78 | P |
| Not spiked | 0 | N | NA | N | 0 | N | NA | N |
|  |  |  |  |  |  |  |  |  |
| **Raw milk** |  |  |  |  |  |  |  |  |
| 10^5^-10^6^ | 6x10^5^ | P | 13.83 | P | 3x10^6^ | P | 19.25 | P |
| 10^4^-10^5^ | 6x10^4^ | P | 17.47 | P | 3x10^5^ | P | 19.50 | P |
| 10^3^-10^4^ | 6x10^3^ | P | 21.31 | P | 3x10^4^ | P | 24.66 | P |
| 10^2^-10^3^ | 6x10^2^ | P | 23.48 | P | 3x10^3^ | P | 31.29 | P |
| 10^1^-10^2^ | 6x10^1^ | N | 27.85 | P | 3x10^2^ | N | 38.71 | P |
| Not spiked | 0 | N | NA | N | 0 | N | NA | N |

* MAP concentrations in spiked milk were derived from colony counts for MAP inoculum plated on M7H9-OADC-MJ agar. ** Chemical decontamination (0.75% HPC for 5 h at room temperature) was applied for raw milks, but not for UHT milks, prior to inoculation of HEYM/ANV slants (Becton Dickinson). Culture result was recorded as Positive (P) if typical MAP colonies were observed after 18 weeks of incubation and Negative (N) if not. *** qPCR result interpreted as per IDEXX RealPCR MAP DNA kit instructions: P, positive (Cq<40), S, suspect (Cq 40 or above), NA, no amplification/negative.

**TABLE 2** MAP status of raw milks from six confirmed MAP-positive cows tested during Milk trial Rounds 1 and 2 and PhMS-qPCR results returned for these naturally infected milks by the six participating laboratories. Presence or absence of viable MAP and MAP DNA was assessed at NEIKER by HPC decontamination and Herrold’s egg yolk ANV medium (HEYM) culture and Adiapure DNA extraction plus IDEXX RealPCR MAP DNA qPCR, respectively. Values in brackets for PCR and PhMS-qPCR represent Cq or mean Cq (if bold font) obtained for duplicate qPCR reactions per sample.

| **Cow ID** | **NEIKER results** | |  | **PhMS-qPCR results** | | | | | | |
| --- | --- | --- | --- | --- | --- | --- | --- | --- | --- | --- |
|  | **HEYM culture*** | **PCR (Mean Cq)**** |  | **Lab 1A** | **Lab 1B** | **Lab 2** | **Lab 3** | **Lab 4** | **Lab 5** | **Lab 6** |
| **Round 1** |  |  |  |  |  |  |  |  |  |  |
| Cow 1 | - | NA |  | NA | NA | NA | NA | NA | NA | NA |
| Cow 2 | - | P (**39.45**) |  | NA | NA | NA | NA | NA | NA | NA |
| Cow 3 | - | NA |  | NA | NA | NA | NA | NA | NA | S (41.83) |
| Cow 4 | - | P (**37.76**) |  | NA | NA | NA | NA | NA | NA | NA |
| Cow 5 | - | P (**38.68**) |  | NA | NA | NA | NA | NA | NA | NA |
| Cow 6 | - | P (**38.79**) |  | NA | S (41.04) | NA | NA | NA | NA | NA |
| Frequency of viable MAP detection | 0/6 |  |  | 0/6 | 0/6 | 0/6 | 0/6 | 0/6 | 0/6 | 0/6 |
|  |  |  |  |  |  |  |  |  |  |  |
| **Round 2** |  |  |  |  |  |  |  |  |  |  |
| Cow 1 | - | NA |  | P (37.35) | NA | NA | S (42.54) | NA | P (38.75) | NA |
| Cow 2 | - | P (**37.40**) |  | NA | NA | NA | NA | NA | NA | NA |
| Cow 3 | - | NA |  | NA | P (38.50) | NA | NA | NA | NA | NA |
| Cow 4 | - | P (**36.95**) |  | NA | P (**38.59**) | NA | NA | NA | NA | NA |
| Cow 5 | - | P (**35.39**) |  | NA | NA | S (42.05) | NA | NA | NA | NA |
| Cow 6 | - | P (**37.80**) |  | NA | P (39.60) | NA | NA | NA | P (37.66) | NA |
| Frequency of viable MAP detection | 0/6 |  |  | 1/6 | 3/6 | 0/6 | 0/6 | 0/6 | 2/6 | 0/6 |

* Milk samples were decontaminated with 0.75% HPC for 5 h before inoculation on HEYM slants. -, no colonies observed after 16 weeks incubation.

** Detecting total MAP DNA and interpreted per IDEXX RealPCR MAP DNA qPCR kit instruction manual: P, positive (Cq<40), S, suspect (Cq 40 or above), NA, no amplification/negative.

**TABLE 3** Confirmation of MAP infection status of (A) artificially spiked faeces (S1 – S6) and (B) naturally infected or non-infected (N1-N12) bovine faeces samples tested during Faeces Trial Round 1 by qPCR and by HPC decontamination and HEYM-VAN culture. NEIKER results were used as Reference Values for determination of PhMS-qPCR test performance characteristics.

**(A) Artificially spiked faeces**

| **Lab** | **Detection method** | **Faeces sample ID, spiking level (CFU MAP/g)** | | | | | |
| --- | --- | --- | --- | --- | --- | --- | --- |
|  |  | **S5, 10^5^** | **S1, 10^4^** | **S3, 10^3^** | **S2, 10^2^** | **S6, 10^1^** | **S4, Not spiked** |
|  |  | Mean Cq of duplicate qPCR reactions per sample | | | | | |
| NEIKER | MagMAX Total nucleic acid isolation kit + IDEXX qPCR | 27.07 | 30.68 | 34.48 | 37.74 | 40.58 | NA |
| ANSES | MagMAX Core nucleic acid purification kit + LSI VetMAX M. paratuberculosis 2.0 qPCR kit | 19.64 | 22.63 | 26.97 | 29.94 | 32.59 | NA |
|  |  |  |  |  |  |  |  |
| NEIKER | HPC decontamination and culture | No result* | No result | No result | No result | No result | No result |
| RAPID-MYCO | HPC decontamination and culture | - | - | + | + | - | - |
| FLI | HPC decontamination and culture | + | -?** | - | -? | No result | No result |

NA, no amplification. +, MAP colonies obtained. -, no growth observed. *Contamination of all slopes inoculated, so MAP growth potentially masked. **-? one culture slant contaminated, two slants no growth, so presence of MAP could not be excluded.

**(B) Naturally infected and non-infected faeces**

| **Lab** | **Detection method** | **MAP positive faeces** | | | | | | | | | | | | | | | | |  | **MAP negative faeces** | | |
| --- | --- | --- | --- | --- | --- | --- | --- | --- | --- | --- | --- | --- | --- | --- | --- | --- | --- | --- | --- | --- | --- | --- |
|  |  | **N11** | **N10** | | **N5** | | **N12** | | **N1** | | | **N7** | | **N3** | | **N8** | | **N9** |  | **N4** | **N2** | **N6** |
|  |  | Mean Cq of duplicate qPCR reactions per sample | | | | | | | | | | | | | | | | | | | | |
| NEIKER | MagMAX Total nucleic acid isolation kit + IDEXX qPCR | 25.26 | | 27.00 | | 27.24 | | 30.31 | | 30.45 | 32.11 | | 32.09 | | 33.37 | | 38.81 | |  | NA | NA | NA |
| ANSES | MagMAX Core nucleic acid purification kit + LSI VetMAX M. paratuberculosis 2.0 qPCR kit | 18.75 | | 20.29 | | 19.75 | | 22.39 | | 21.39 | 23.15 | | 23.64 | | 25.73 | | 35.97 | |  | NA | NA | NA |
|  |  |  | |  | |  | |  | |  |  | |  | |  | |  | |  |  |  |  |
| NEIKER | HPC decontamination and HEYM culture | + | | + | | + | | + | | + | + | | + | | + | | - | |  | - | - | - |
| RAPID-MYCO | HPC decontamination and HEYM culture | + | | + | | + | | + | | + | + | | + | | + | | - | |  | - | - | - |
| FLI | HPC decontamination and HEYM culture | + | | + | | + | | No result* | | No result | + | | + | | + | | No result | |  | No result | -? ** | - |

NA, no amplification. + isolation of MAP colony or colonies confirmed, - no growth observed. *Contamination of all slants inoculated, so MAP growth potentially masked. **-? one culture slant contaminated, two slants no growth, so presence of MAP could not be excluded.

**TABLE 4** MAP status of naturally infected faeces tested during Faeces Trial Round 2 confirmed at NEIKER, assessed by DNA extraction and qPCR (three different methods performed in two laboratories) and HPC decontamination plus HEYM culture (performed in three laboratories). NA, no amplification; + confirmed MAP isolate obtained, - no MAP growth recorded.

|  | | | | | | | | | | |  | | | | | | | | | |  |
| --- | --- | --- | --- | --- | --- | --- | --- | --- | --- | --- | --- | --- | --- | --- | --- | --- | --- | --- | --- | --- | --- |
| **Lab** | **Detection method** | **MAP positive faeces** | | | | | | | | | **MAP negative faeces** | | | | | | | | | |  |
|  |  | **N11** | **N12** | **N8** | **N4** | **N5** | **N14** | **N17** | **N15** | **N18** |  | **N1** | **N2** | **N3** | **N6** | **N7** | **N9** | **N10** | **N13** | **N16** |  |
|  |  | Mean Cq of duplicate qPCR reactions per sample | | | | | | | | | | | | | | | | | | | |
| NEIKER | MagMAX Total nucleic acid isolation kit + IDEXX qPCR | 25.24 | 27.05 | 26.97 | 29.35 | 30.00 | 31.39 | 31.72 | 33.42 | 34.63 |  | NA | NA | NA | NA | NA | NA | NA | NA | NA |  |
| NEIKER | MagMAX Total nucleic acid isolation kit + NEIKER ISMAP02 qPCR | 23.67 | 25.20 | 25.96 | 23.96 | 27.55 | 27.93 | 27.78 | 30.31 | 30.78 |  | NA | NA | NA | NA | NA | NA | NA | NA | NA |  |
| ANSES | MagMAX Core nucleic acid purification kit + LSI VetMAX M. paratuberculosis 2.0 qPCR kit | 19.92 | 20.84 | 21.86 | 22.83 | 24.04 | 24.64 | 24.39 | 26.75 | 27.58 |  | NA | NA | NA | NA | NA | NA | 38.81 | NA | 37.59 |  |
|  |  |  |  |  |  |  |  |  |  |  |  |  |  |  |  |  |  |  |  |  |  |
| NEIKER | HPC decontamination and HEYM culture | + | + | + | + | + | + | + | + | + |  | - | - | - | - | - | - | - | - | - |  |
| RAPID-MYCO | HPC decontamination and HEYM culture | + | + | + | + | + | + | - | + | + |  | - | - | - | - | - | - | - | - | - |  |
| FLI | HPC decontamination and HEYM culture | + | + | + | + | + | + | + | + | + |  | - | - | - | - | - | - | - | - | - |  |

**TABLE 5**. Details of the equipment used in each participating laboratory to perform the PhMS-qPCR assay.

| **Lab ID** | **Sonication** | **Magnetic separation** | **qPCR** |
| --- | --- | --- | --- |
| 1A | Fisherbrand P30H (Fisher Scientific) | Kingfisher 1 mL (Thermofisher) | PCRMax Eco™ 48 Real PCR (Cole Parmer) |
| 1B | Fisherbrand P30H (Fisher Scientific) | PurePrep 24D (MolGenGEN BV) | PCRMax Eco™ 48 Real PCR (Cole Parmer) |
| 2 | Elmasonic P30H (Elma Schmidbauer Gmbh) | Kingfisher Flex (Thermofisher) | QuantStudio 5, Applied Biosystems (Thermo Fisher Scientific) |
| 3 | Ultrasons (40 Hz) (J.P. Selecta) | Kingfisher Flex (Thermofisher) | CFX96 Real-Time System (Bio-Rad Laboratories) |
| 4 | Elma D-78224 (Elma Schmidbauer Gmbh) | Kingfisher DUO PRIME (Thermofisher) | C1000 Touch Thermal Cycler (CFX96 Real-Time System) (Bio-Rad Laboratories) |
| 5 | SONOREX (Bandelin Electronics Gmbh) | Magnetic rack for 1.5 ml tubes | Light Cycler 480 (Roche) |
| 6 | SONOREX Super RK 100 H (Bandelin Electronics Gmbh) | Magnetic rack for 1.5 ml tubes | Applied Biosystems 7500 Real Time PCR System (Thermo Fisher Scientific) |

| **TABLE 6.** Reproducibility of the PhMS-qPCR assay performed in six different laboratories to test (A) MAP spiked raw milk and (B) MAP spiked UHT milk. Binary outcome data from Milk Trial rounds 1 and 2 were combined for each sample matrix to calculate Cohen’s Kappa agreement between pairs of laboratories. Significance of agreement: ^NS^ p>0.05, * p<0.05, ** p<0.01, *** p<0.001. | | | | | | | | | | |
| --- | --- | --- | --- | --- | --- | --- | --- | --- | --- | --- |
| 1. **MAP-spiked raw cows’ milk** | | | | | |  | |  | |  |
| **Lab pairs** | **2 x 2 contingency table** | | | | **McNemar's Chi^2^ p-value** | | **Agreement (%)** | | **Cohen's Kappa**  **(95% limits)** | |
|  | **(+/+)** | **(+/-)** | **(-/+)** | **(-/-)** |  |  |  |  |  |  |
| Lab 1A, Lab 1B | 10 | 0 | 1 | 1 | 0.0098** | | 91.7 | | 0.6250 (-0.0273, 1.2773) | |
| Lab 1A, Lab 2 | 10 | 0 | 0 | 2 | 0.0005*** | | 100.0 | | 1.0000 (1.0000, 1.0000) | |
| Lab 1A, Lab 3 | 8 | 2 | 0 | 2 | 0.0142* | | 83.3 | | 0.5714 (0.0815, 1.0613) | |
| Lab 1A, Lab 4 | 8 | 2 | 0 | 2 | 0.0142* | | 83.3 | | 0.5714 (0.0815, 1.0613) | |
| Lab 1A, Lab 5 | 8 | 2 | 1 | 1 | 0.1855^NS^ | | 75.0 | | 0.2500 (-0.3716, 0.8716) | |
| Lab 1A, Lab 6 | 8 | 2 | 0 | 2 | 0.0142* | | 83.3 | | 0.5714 (0.0815, 1.0613) | |
| Lab 1B, Lab 2 | 10 | 0 | 0 | 2 | 0.0005*** | | 100.0 | | 1.0000 (1.0000, 1.0000) | |
| Lab 1B, Lab 3 | 8 | 3 | 0 | 1 | 0.0698^NS^ | | 75.0 | | 0.3077 (-0.1819, 0.7973) | |
| Lab 1B, Lab 4 | 8 | 3 | 0 | 1 | 0.0698^NS^ | | 75.0 | | 0.3077 (-0.1819, 0.7973) | |
| Lab 1B, Lab 5 | 9 | 2 | 0 | 1 | 0.0352* | | 83.3 | | 0.4286 (-0.1647, 1.0219) | |
| Lab 1B, Lab 6 | 8 | 3 | 0 | 1 | 0.0698^NS^ | | 75.0 | | 0.3077 (-0.1819, 0.7973) | |
| Lab 2, Lab 3 | 8 | 2 | 0 | 2 | 0.0142* | | 83.3 | | 0.5714 (0.0815, 1.0613) | |
| Lab 2, Lab 4 | 8 | 2 | 0 | 2 | 0.0142* | | 83.3 | | 0.5714 (0.0815, 1.0613) | |
| Lab 2, Lab 5 | 8 | 2 | 1 | 1 | 0.1855^NS^ | | 75.0 | | 0.2500 (-0.3716, 0.8716) | |
| Lab 2, Lab 6 | 8 | 2 | 0 | 2 | 0.0142* | | 83.3 | | 0.5714 (0.0815, 1.0613) | |
| Lab 3, Lab 4 | 8 | 0 | 0 | 4 | 0.0003*** | | 100.0 | | 1.0000 (1.0000, 1.0000) | |
| Lab 3, Lab 5 | 8 | 0 | 1 | 3 | 0.0023*** | | 91.7 | | 0.8000 (0.4323,1.1677) | |
| Lab 3, Lab 6 | 8 | 0 | 0 | 4 | 0.0003*** | | 100.0 | | 1.0000 (1.0000, 1.0000) | |
| Lab 4, Lab 5 | 8 | 0 | 1 | 3 | 0.0023*** | | 91.7 | | 0.8000 (0.4323,1.1677) | |
| Lab 4, Lab 6 | 8 | 0 | 0 | 4 | 0.0003*** | | 100.0 | | 1.0000 (1.0000, 1.0000) | |
| Lab 5, Lab 6 | 8 | 1 | 0 | 3 | 0.0023*** | | 91.7 | | 0.8000 (0.4323,1.1677) | |

| 1. **MAP-spiked UHT milk** | | | |  |  |  |  |
| --- | --- | --- | --- | --- | --- | --- | --- |
| **Lab pairs** | **2 x 2 contingency table** | | | | **McNemar's Chi^2^ p-value** | **Agreement (%)** | **Cohen's Kappa  (95% limits)** |
|  | **(+/+)** | **(+/-)** | **(-/+)** | **(-/-)** |  |  |  |
| Lab 1A, Lab 1B | 10 | 0 | 1 | 1 | 0.0098** | 91.7 | 0.6250 (-0.0273, 1.2773) |
| Lab 1A, Lab 2 | 9 | 1 | 0 | 2 | 0.0036** | 91.7 | 0.7500 (0.2958, 1.2042) |
| Lab 1A, Lab 3 | 8 | 2 | 0 | 2 | 0.0142* | 83.3 | 0.5714 (0.0815, 1.0613) |
| Lab 1A, Lab 4 | 9 | 1 | 0 | 2 | 0.0036** | 91.7 | 0.7500 (0.2958,1.2042) |
| Lab 1A, Lab 5 | 8 | 2 | 0 | 2 | 0.0142* | 83.3 | 0.5714 (0.0815, 1.0613) |
| Lab 1A, Lab 6 | 8 | 2 | 0 | 2 | 0.0142* | 83.3 | 0.5714 (0.0815, 1.0613) |
| Lab 1B, Lab 2 | 9 | 2 | 0 | 1 | 0.0352* | 83.3 | 0.4286 (-0.1647, 1.0219) |
| Lab 1B, Lab 3 | 8 | 3 | 0 | 1 | 0.0698^NS^ | 75.0 | 0.3077 (-0.1819, 0.7973) |
| Lab 1B, Lab 4 | 10 | 1 | 0 | 1 | 0.0098** | 91.7 | 0.6250 (-0.0273, 1.2773) |
| Lab 1B, Lab 5 | 8 | 3 | 0 | 1 | 0.0698^NS^ | 75.0 | 0.3077 (-0.1819, 0.7973) |
| Lab 1B, Lab 6 | 8 | 3 | 0 | 1 | 0.0698^NS^ | 75.0 | 0.3077 (-0.1819, 0.7973) |
| Lab 2, Lab 3 | 8 | 1 | 0 | 3 | 0.0023** | 91.7 | 0.8000 (0.4323,1.1677) |
| Lab 2, Lab 4 | 9 | 0 | 1 | 2 | 0.0036** | 91.7 | 0.7500 (0.2958,1.2042) |
| Lab 2, Lab 5 | 8 | 1 | 0 | 3 | 0.0023** | 91.7 | 0.8000 (0.4323,1.1677) |
| Lab 2, Lab 6 | 8 | 1 | 0 | 3 | 0.0023** | 91.7 | 0.8000 (0.4323,1.1677) |
| Lab 3, Lab 4 | 8 | 0 | 2 | 2 | 0.0142* | 83.3 | 0.5714 (0.0815, 1.0613) |
| Lab 3, Lab 5 | 8 | 0 | 0 | 4 | 0.0003*** | 100.0 | 1.0000 (1.0000, 1.0000) |
| Lab 3, Lab 6 | 8 | 0 | 0 | 4 | 0.0003*** | 100.0 | 1.0000 (1.0000, 1.0000) |
| Lab 4, Lab 5 | 8 | 2 | 0 | 2 | 0.0142* | 83.3 | 0.5714 (0.0815, 1.0613) |
| Lab 4, Lab 6 | 8 | 2 | 0 | 2 | 0.0142* | 83.3 | 0.5714 (0.0815, 1.0613) |
| Lab 5, Lab 6 | 8 | 0 | 0 | 4 | 0.0003*** | 100.0 | 1.0000 (1.0000, 1.0000) |

**TABLE 7** Reproducibility of PhMS-qPCR assay performed in six different laboratories to test faeces from confirmed Johne’s disease positive or negative cattle: (A) Round 1, 12 samples and (B) Round 2, 18 samples. During Round 1, 1 ml clarified faecal supernatant was tested by all laboratories except Lab 1B (5 ml). During Round 2, 5 ml clarified supernatant was tested by all laboratories. Binary outcome data for each Round were used to calculate Cohen’s Kappa agreement between pairs of laboratories. Significance of agreement: ^NS^ p>0.05, * p<0.05, ** p<0.01, *** p<0.001.

| 1. **FAECES TRIAL, ROUND 1** | | | | | | | | | | |
| --- | --- | --- | --- | --- | --- | --- | --- | --- | --- | --- |
| **Lab pairs** | **2 x 2 contingency table** | | | | **McNemar's Chi^2^ p-value** | **Agreement (%)** | | **Cohen's Kappa  (95% limits)** |  |  |
|  | **(+/+)** | **(+/-)** | **(-/+)** | **(-/-)** |  |  |  |  |  |  |
| Lab 1A, Lab 1B | 2 | 0 | 7 | 3 | 0.1855^NS^ | 41.7 | 0.1250 (-0.0776, 0.3276) | | |  |
| Lab 1A, Lab 2 | 2 | 0 | 5 | 5 | 0.0952^NS^ | 58.3 | 0.2500 (-0.0821, 0.5821) | | |  |
| Lab 1A, Lab 3 | 1 | 1 | 4 | 6 | 0.3967^NS^ | 58.3 | 0.0625 (-0.4140, 0.5390) | | |  |
| Lab 1A, Lab 4 | 1 | 1 | 2 | 8 | 0.1855^NS^ | 75.0 | 0.2500 (-0.3716, 0.8716) | | |  |
| Lab 1A, Lab 5 | 2 | 0 | 5 | 5 | 0.0952^NS^ | 58.3 | 0.2500 (-0.0821, 0.5821) | | |  |
| Lab 1A, Lab 6 | 2 | 0 | 4 | 6 | 0.0607^NS^ | 66.7 | 0.3333 (-0.0643, 0.7309) | | |  |
| Lab 1B, Lab 2 | 5 | 4 | 2 | 1 | 0.3677^NS^ | 50.0 | -0.0909 (-0.6042, 0.4223) | | |  |
| Lab 1B, Lab 3 | 3 | 6 | 2 | 1 | 0.1562^NS^ | 33.3 | -0.2308 (-0.6993,0.2377) | | |  |
| Lab 1B, Lab 4 | 1 | 8 | 2 | 1 | 0.0271* | 16.7 | -0.3333 (-0.8123,0.1517) | | |  |
| Lab 1B, Lab 5 | 5 | 4 | 2 | 1 | 0.3677^NS^ | 50.0 | -0.0909 (-0.6042, 0.4223) | | |  |
| Lab 1B, Lab 6 | 4 | 5 | 2 | 1 | 0.2525^NS^ | 41.7 | -0.1667 (-0.6498, 0.3165) | | |  |
| Lab 2, Lab 3 | 5 | 2 | 0 | 5 | 0.0067** | 83.3 | 0.6757 (0.2875, 1.0638) | | |  |
| Lab 2, Lab 4 | 3 | 4 | 0 | 5 | 0.0455* | 66.7 | 0.3846 (-0.0035, 0.7727) | | |  |
| Lab 2, Lab 5 | 7 | 0 | 0 | 5 | 0.0003*** | 100.0 | 1.0000 (1.0000, 1.0000) | | |  |
| Lab 2, Lab 6 | 5 | 2 | 1 | 4 | 0.0395* | 75.0 | 0.5000 (0.0169, 0.9831) | | |  |
| Lab 3, Lab 4 | 3 | 2 | 0 | 7 | 0.0090** | 83.3 | 0.6364 (0.2078, 1.6490) | | |  |
| Lab 3, Lab 5 | 5 | 0 | 2 | 5 | 0.0067** | 83.3 | 0.6757 (0.2875, 1.0638) | | |  |
| Lab 3, Lab 6 | 4 | 1 | 1 | 6 | 0.0114* | 83.3 | 0.6571 (0.2242, 1.0901) | | |  |
| Lab 4, Lab 5 | 3 | 4 | 0 | 5 | 0.0455* | 66.7 | 0.3846 (-0.0035, 0.7727) | | |  |
| Lab 4, Lab 6 | 3 | 0 | 3 | 6 | 0.0288* | 75.0 | 0.5000 (0.0757, 0.9243) | | |  |
| Lab 5, Lab 6 | 5 | 2 | 1 | 4 | 0.0395* | 75.0 | 0.5000 (0.0169, 0.9831) | | |  |

| 1. **FAECES TRIAL, ROUND 2** | | | | | | | |
| --- | --- | --- | --- | --- | --- | --- | --- |
| **Lab pairs** | **2 x 2 contingency table** | | | | **McNemar's Chi^2^ p-value** | **Agreement (%)** | **Cohen's Kappa  (95% limits)** |
|  | **(+/+)** | **(+/-)** | **(-/+)** | **(-/-)** |  |  |  |
| Lab 1A, Lab 1B | 4 | 4 | 4 | 6 | 0.3357^NS^ | 55.6 | 0.1000(-0.3606, 0.5606) |
| Lab 1A, Lab 2 | 5 | 3 | 3 | 7 | 0.0840^NS^ | 66.7 | 0.3250 (-0.1141, 0.7641) |
| Lab 1A, Lab 3 | 4 | 4 | 3 | 7 | 0.1935^NS^ | 61.1 | 0.2025 (-0.2504, 0.6555) |
| Lab 1A, Lab 4 | 5 | 3 | 3 | 7 | 0.0840^NS^ | 66.7 | 0.3250 (-0.1141, 0.7641) |
| Lab 1A, Lab 5 | 5 | 3 | 5 | 5 | 0.2979^NS^ | 55.6 | 0.1220 (-0.3242, 0.5681) |
| Lab 1A, Lab 6 | 5 | 3 | 4 | 6 | 0.1714^NS^ | 61.1 | 0.2222 (-0.2254, 0.6699) |
| Lab 1B, Lab 2 | 7 | 1 | 1 | 9 | 0.0005*** | 88.9 | 0.7750 (0.4811, 1.0689) |
| Lab 1B, Lab 3 | 6 | 2 | 1 | 9 | 0.0025** | 83.3 | 0.6582 (0.3080, 1.0084) |
| Lab 1B, Lab 4 | 7 | 1 | 1 | 9 | 0.0005*** | 88.9 | 0.7750 (0.4811, 1.0689) |
| Lab 1B, Lab 5 | 8 | 0 | 2 | 8 | 0.0003*** | 88.9 | 0.7805 (0.5007, 1.0603) |
| Lab 1B, Lab 6 | 7 | 1 | 2 | 8 | 0.0022** | 83.3 | 0.6667 (0.3245, 1.0089) |
| Lab 2, Lab 3 | 6 | 2 | 1 | 9 | 0.0025** | 83.3 | 0.6582 (0.3080,1.0084) |
| Lab 2, Lab 4 | 7 | 1 | 1 | 9 | 0.0005*** | 88.9 | 0.7750 (0.4811, 1.0689) |
| Lab 2, Lab 5 | 8 | 0 | 2 | 8 | 0.0003*** | 88.9 | 0.7805 (0.5007, 1.0603) |
| Lab 2, Lab 6 | 8 | 0 | 1 | 9 | 0.0001*** | 94.4 | 0.8889 (0.6786, 1.0992) |
| Lab 3, Lab 4 | 7 | 0 | 1 | 10 | 0.0001*** | 94.4 | 0.8861 (0.6705, 1.1017) |
| Lab 3, Lab 5 | 7 | 0 | 3 | 8 | 0.0012** | 83.3 | 0.6747 (0.3569, 0.9925) |
| Lab 3, Lab 6 | 6 | 1 | 3 | 8 | 0.0018** | 77.8 | 0.5556 (0.1810, 0.9301) |
| Lab 4, Lab 5 | 8 | 0 | 2 | 8 | 0.0003*** | 88.9 | 0.7805 (0.5007, 1.0603) |
| Lab 4, Lab 6 | 7 | 1 | 2 | 8 | 0.0022** | 83.3 | 0.6667 (0.3245, 1.0089) |
| Lab 5, Lab 6 | 8 | 1 | 0 | 9 | 0.0001*** | 94.4 | 0.8889 (0.6786, 1.0992) |
